# Beyond motor recovery: emerging perspectives in the management of peripheral facial palsy—a narrative review

**DOI:** 10.3389/fneur.2026.1915135

**Published:** 2026-09-07

**Authors:** Harry von Piekartz, Anna-Maria Kuttenreich, Bernhard Taxer

**Affiliations:** 1 Physical Therapy and Rehabilitation Science, Hochschule Osnabrück, Fakultät Wirtschafts- und Sozialwissenschaften, Osnabrück, Germany; 2 Practice for Orofacial Physiotherapy and Applied Pain Sciences, Ootmarsum, Netherlands; 3 Kommunikationsreich Kuttenreich, Speech and Language Therapy, Ingolstadt, Germany; 4 Department of Neurology, Neurocritical Care, and Neurorehabilitation, Centre for Cognitive Neuroscience, Paracelsus Medical University, Member of ERN EpiCARE, Christian Doppler University Hospital, Salzburg, Austria

**Keywords:** body representation, facial rehabilitation, neurocognitive rehabilitation, peripheral facial palsy, physiotherapy, sensorimotor integration, somatosensory dysfunction, speech and language therapy

## Abstract

**Background:**

Peripheral facial palsy (PFP) is a common cranial nerve disorder that may result in persistent impairments extending beyond facial muscle weakness, including facial asymmetry, synkinesis, altered emotional expression, and reduced quality of life. Traditional rehabilitation approaches have predominantly focused on motor recovery and facial muscle function. However, emerging evidence suggests that somatosensory processing, body awareness, body image, and sensorimotor integration may play a more substantial role in recovery and long-term outcomes than previously recognized.

**Aims and methods:**

This narrative review aims to synthesize current evidence regarding multimodal rehabilitation strategies for PFP, with particular emphasis on craniofacial somatosensory disturbances and their clinical implications. The literature was reviewed across three domains: (A) peripheral facial palsy and related diagnoses, (B) assessment of facial somatosensory distortion and body representation, and (C) interventions targeting somatosensory dysfunction. In addition to established clinical examinations and patient-reported outcome measures, emerging assessment strategies addressing body representation, self-perception, and sensory discrimination are discussed.

**Results:**

The review highlights growing evidence supporting the integration of somatosensory and neurocognitive approaches into clinical management. Altered facial perception and disrupted sensorimotor integration may contribute to persistent dysfunction and reduced participation. To address these mechanisms, a structured neurocognitive rehabilitation framework, the 8-E model, is presented. Furthermore, targeted somatosensory interventions, including two-point discrimination training, auditory acuity training, and trigeminal blink reflex training, and pre- and post-lesion experience using autobiographical memory are proposed to enhance sensory discrimination, cortical representation, and sensorimotor integration.

**Conclusion:**

Current findings suggest that a broader biopsychosocial and neurocognitive perspective may complement conventional motor rehabilitation in PFP. Future research should investigate the clinical effectiveness of somatosensory-based interventions and further clarify their role in optimizing functional recovery, participation, and quality of life.

## Introduction

Peripheral facial palsy (PFP) is a prevalent cranial nerve disorder typified by acute weakness or paralysis of the facial musculature due to dysfunction of the facial nerve (1). Although a significant proportion of patients exhibit spontaneous recovery, a considerable subgroup experiences persistent sequelae, including facial asymmetry, stiffness, reduced movement selectivity, synkinesis, and impaired emotional expressivity (2). Given that facial movements are central to social communication, emotional signaling, and self-perception, the impact of PFP extends far beyond motor impairment alone. Patients frequently report feelings of embarrassment, social withdrawal, anxiety, and a reduced quality of life, particularly when facial expression remains distorted during activities such as speaking, smiling, or blinking (3).

The prevailing conceptualization of PFP as a peripheral nerve disorder has historically informed the focus of rehabilitation interventions. These interventions have centered on the restoration of facial symmetry, the promotion of peripheral motor recovery, and the prevention of contracture or maladaptive co-contraction (4). However, this peripheral perspective is incomplete. The facial nerve represents the final common pathway of a broader sensorimotor–emotional network that includes the facial nucleus in the brainstem, corticobulbar tracts, primary motor and somatosensory cortices, supplementary motor areas, and limbic structures involved in emotional processing (5). Consequently, facial expression cannot be considered a mere mechanical output of muscle activation; rather, it is a neurobiological process that integrates intention, sensory feedback, motor planning and emotional meaning (6).

This dual organization helps to explain why facial palsy can disrupt not only movement amplitude and coordination, but also the naturalness and perceived authenticity of emotional expression (7). In healthy individuals, continuous refinement of facial movements is achieved through afferent sensory input from the trigeminal and proprioceptive pathways (5). These pathways convey information regarding muscle length, tension, and cutaneous deformation to central motor networks. In the event of nerve injury, the alteration of this feedback loop results in the brain being compelled to adapt to incomplete or distorted sensory information, which can lead to changes in motor output and self-perception (8).

The pathophysiology of PFP is heterogeneous and may involve viral inflammation, compression, trauma, or iatrogenic nerve damage, resulting in neurapraxia, axonal degeneration, and subsequent regeneration (9). However, it is important to note that during the recovery phase, regeneration does not guarantee the restoration of normal function. Aberrant reinnervation, incomplete remyelination, swelling of the nerve trunk with increased nociceptive input, and altered central control can all contribute to abnormal co-contractions, hypertonicity, and the development of synkinesis (10). Synkinesis is defined as the involuntary contraction of one facial muscle group during the voluntary activation of another. A case in point would be the excessive occurrence of eye closure during smiling, or mouth contraction during blinking. These motor abnormalities are not solely peripheral consequences of nerve regeneration but are increasingly recognized as manifestations of maladaptive neuroplasticity within facial motor networks of the central nervous system (5, 11).

Neuroimaging findings support this concept. Klingner et al. (12) demonstrated that Bell’s palsy is associated with cortical reorganization, suggesting that facial nerve dysfunction is accompanied by altered cortical representation and sensorimotor processing. In a later report, these authors described the time course of cortical plasticity following facial nerve palsy, suggesting that these changes develop dynamically during recovery rather than remaining static (13). Similarly, studies on facial synkinesis have demonstrated patterns of cortical reorganization and altered functional connectivity, supporting the idea that persistent facial dysfunction reflects both peripheral and central mechanisms. This is clinically important because it suggests that assessment and treatment should not be limited to the peripheral nerve or isolated muscles, but should also address the brain’s adaptive and maladaptive responses to altered facial input (14, 15).

### Somatosensory distortion in peripheral facial palsy

Somatosensory distortion refers to a discrepancy between a person’s perception of their body posture, body size, or body movement and the body’s actual physical state (16). In peripheral facial palsy, patients often report that the affected half of the face feels numb, heavy, detached, or “swollen,” even though standard sensory tests do not indicate a significant loss of trigeminal or cutaneous sensation (17). This is further corroborated by a subsequent prospective QST (Quantitative Sensory Testing) study from the same research group that had previously reported cortical reorganization in Bell’s palsy (12, 13), which found no significant differences in somatosensory function between the paretic and non-paretic side (18). This dissociation between preserved peripheral sensory function and central cortical changes underscores that the functional and clinical significance of cortical reorganization in PFP remains incompletely understood. Discrepancies like this suggest that the subjective disturbance may not solely stem from peripheral deafferentation, but rather from a mismatch between facial motor intention and the absence or altered quality of somatosensory feedback from the face. Cortical reorganization following facial nerve palsy can shift or blur the somatosensory representation of the face, potentially leading to less accurate cortical maps and disrupted integration of facial proprioception and touch (19). As a result, patients may perceive their own facial expressions as inaccurate or “inappropriate,” even though observers rate them as relatively appropriate. This can lead to hesitant facial movements, increased facial movements without relaxation phases, and a tendency to under- or over-activate facial muscles while making expressions. This persistent sensorimotor mismatch can hinder the refinement of facial muscle control, delay the recovery of coordinated facial movements, and ultimately affect the precision of visual emotional expression and the ability to recognize and interpret facial emotions in others (18).

In addition to these perceptual changes, other sensory functions in which trigeminal–facial circuits play a central role are frequently disturbed. The blink reflex, which is mediated by the trigeminal nerve, is frequently found to be abnormal in cases of acute peripheral facial palsy (20). The blink reflex may exhibit several abnormalities, including reduced symmetry or absence on the affected side. Such alterations in the automatic blink response are particularly observed in patients who later develop post-paralytic facial synkinesis (21). These findings indicate reorganization within the brainstem and a disturbance of the “gating” between sensory input and motor output, so that part of the normal coupling between feeling and moving in the face becomes functionally uncoupled. This finding is consistent with patients’ reports of an altered sense of facial normality, ownership, or reliability. In the authors’ clinical experience, such perceptual disturbances are frequently reported by individuals with chronic facial dysfunction.

Furthermore, auditory processing may also be affected. The involvement of the facial nerve proximal to the stapedius nerve has been shown to reduce or abolish the acoustic (stapedius) reflex (22). This, in turn, has been demonstrated to diminish the normal attenuation of loud sounds and can result in hyperacusis or sound intolerance on the affected side, despite normal pure-tone thresholds (23).

In Bell’s palsy, studies have demonstrated the absence of stapedius reflexes and the presence of reduced discomfort thresholds for loud sounds on the affected side. This suggests a subclinical change in auditory acuity, which patients may experience as “too loud,” “sharp,” or “uncomfortable” rather than as hearing loss (24). Collectively, these alterations in the physiology of the blink reflex and auditory modulation emphasize, that peripheral facial palsy is not merely a motor disorder, but rather, it is accompanied by a more extensive disruption of multimodal sensorimotor processing. This disruption can exacerbate somatosensory distortion and functional limitations (25).

### Body image, body schema and sensorimotor discrepancy

Body image and body schema are complementary concepts that determine how people perceive not only their bodies but also their faces. As posited by Sattin et al. (26), body schema can be defined as the unconscious, multimodal representation of body structure and movement used for real-time motor control. In contrast, body image can be defined as the conscious, visual and emotional representation of appearance and self-worth. In cases of facial palsy, altered afferent input from the affected side has been shown to disrupt the body schema, causing patients to misjudge the symmetry, intensity, or timing of their facial movements, even when the visible expression is relatively adequate (27). Concurrently, the highly visible nature of the face exerts a substantial influence on body image, with persistent asymmetry or inhibition of the facial muscles contributing to a negative self-image, avoidance of mirrors, and reduced social participation. Some patients also report appearance-related stress, exhibiting characteristics reminiscent of body dysmorphic concerns (28). This combination of a distorted body schema and an impaired body image can perpetuate a persistent sensorimotor mismatch, in which intended movement, experienced movement, and perceived facial expression do not align, thereby further reinforcing maladaptive motor patterns and central reorganization (6).

**Figure 1:**
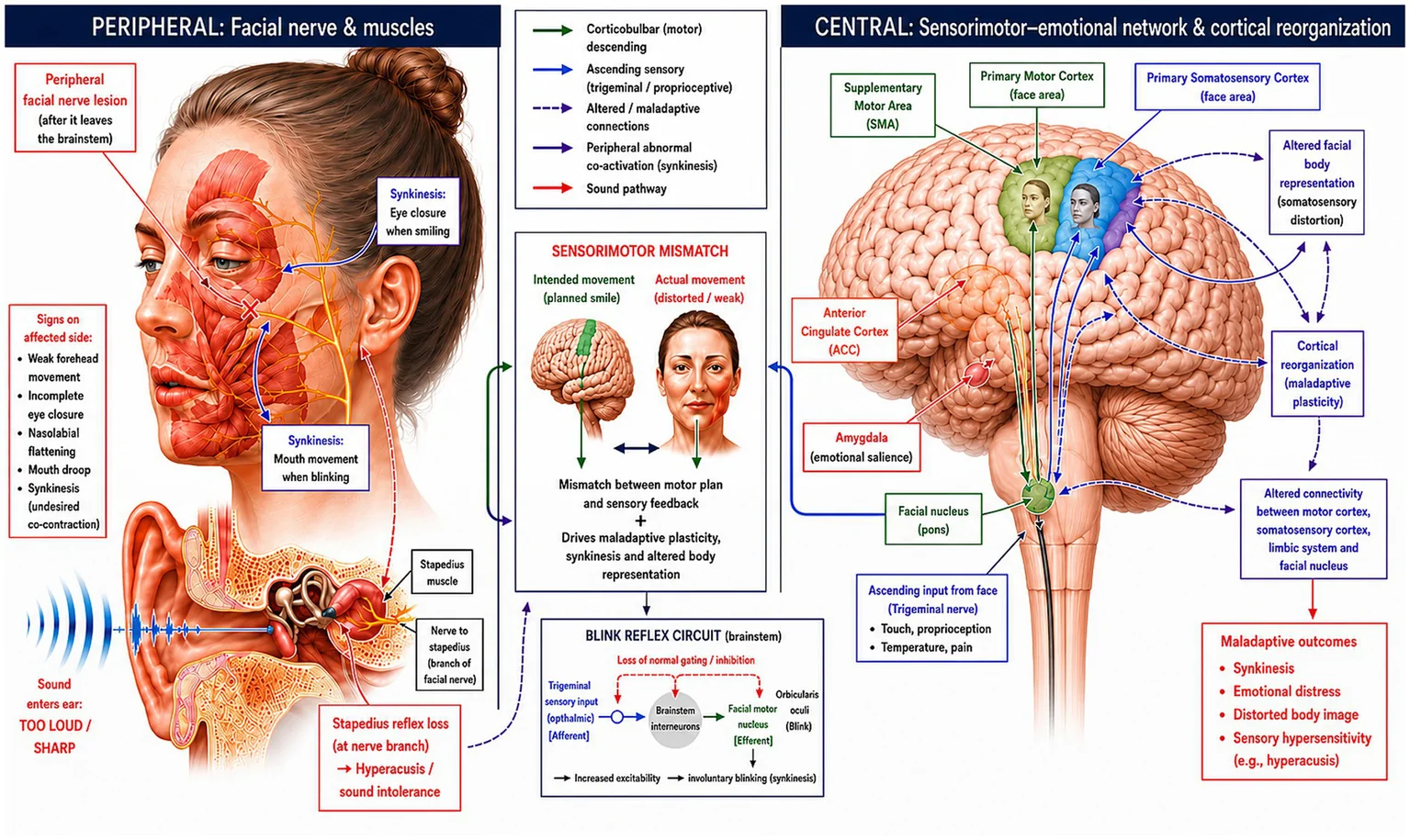
Peripheral and central mechanisms in peripheral facial palsy. Schematic overview of how a peripheral lesion of the facial nerve leads to facial weakness, synkinesis and loss of the stapedius reflex with hyperacusis (left), while altered sensory input from the face drives sensorimotor mismatch, disturbed blink reflex modulation and maladaptive cortical reorganization within facial sensorimotor and limbic networks (right). Together, these changes contribute to somatosensory distortion of the face, abnormal emotional expressivity and broader multimodal hypersensitivity rather than a purely peripheral motor deficit (figure was developed by H. von Piekartz using a digital illustration tool, based on the theoretical framework outlined in the introduction).

In this context, disruptions in emotional-cognitive functioning, as well as changes in (facial) body image and body schema, appear particularly relevant (29). As demonstrated in the research by Cavallaro et al. (30), Calabrò et al. (31), and Gervasoni et al. (32), specific therapeutic techniques, including structured emotional-facial training, remote cognitive therapeutic exercises, and motor imagery-based programs, have been shown to effectively target the patient’s attention towards facial expressions, their emotional significance, and the cognitive representation of facial movements. The authors believe that by guiding patients in identifying and mentally rehearsing facial expressions prior to their execution, these interventions can facilitate the realignment of the link between intention, sensory feedback, and motor output. This, in turn, can support adaptive sensorimotor integration and recalibration of the distorted body schema. Moreover, by emphasizing the emotional and communicative function of facial expressions as opposed to purely mechanical symmetry, emotion-cognitive training has the potential to address concerns regarding body image and the psychosocial impact of facial palsy. Such strategies are in alignment with emerging neurorehabilitation models that integrate techniques specific to the body schema and body image (e.g., action observation, motor imagery, and sensorimotor retraining) into treatment protocols for conditions involving impaired sensorimotor and emotional integration (3). For a schematic visual overview, see Figure 1.

In the recent literature on rehabilitation for peripheral facial palsy, many interventions are primarily based on hands-off strategies, such as exercise therapy, mirror or EMG biofeedback, and remote or app-based neurocognitive programs (4). Concurrently, clinical protocols developed within specialized physical therapy and speech-language pathology disciplines frequently integrate a hands-off training approach with hands-on components, encompassing manual soft-tissue techniques, stretching exercises, and guided facilitation of orofacial movements. Illustrative examples of this integration can be observed in mime therapy and related neuromuscular re-education programs (33). The utilization of integrated methodologies is designed to exert influence on orofacial somatosensory disruption and neuromuscular coordination, in addition to modulating underlying emotional and cognitive processes. This is achieved by the incorporation of facial movement practice within a more extensive framework encompassing communication, self-perception, and attention control (32).

### Hands on and hands off

Drawing upon a foundation of clinical experience and recent evidence, the authors assert that the integration of “hands-on” interventions, encompassing tactile stimulation, neurodynamic techniques, stretching exercises, and manual facilitation, with “hands-off” approaches, such as structured facial exercise therapy, mirror or EMG biofeedback, lateralization training, motor visualization, emotion-focused cognitive training, and mindful movement, constitutes a conceptually tenable strategy for addressing persistent somatosensory, cognitive, and emotional disorders in facial palsy. This assertion is substantiated by evidence supporting cortical reorganization and alterations in the sensorimotor network during the recovery process (25). However, the extant evidence remains circumscribed. A review of the literature on physical therapy, speech-language pathology, and exercise-based rehabilitation for facial palsy reveals several key findings (33). Firstly, the review highlights the heterogeneity of study designs, small sample sizes, and a predominance of low- to moderate-quality studies, with few robust studies directly evaluating combined manual and neurocognitive approaches. Secondly, it is evident that the role of somatosensory disintegration and its interaction with central brain processes has not yet been fully explored in the literature on physical therapy or speech-language pathology, despite this line of research, which links somatosensory modulation, neuroplasticity, and emotion-cognitive training, being clearly on the rise (30, 33).

Based on the above, the following paragraphs summarize current interventions for peripheral facial nerve palsy that integrate somatosensory-oriented techniques with brain-focused assessment and rehabilitation approaches. The present article focuses on approaches that integrate hands-on orofacial therapy and sensory retraining with emotional training, motor visualization, and other neurocognitive or telerehabilitation methods during assessment and treatment. As a narrative review it provides an overview of the emerging evidence and discusses the proposed underlying mechanisms. It furthermore outlines potential future directions for research in neurorehabilitation.

Given the current scarcity of mechanistic and interventional studies conducted specifically in PFP populations, this review draws in part on concepts established in related fields such as chronic pain and complex regional pain syndrome (CRPS) research, including body schema and body image disturbances and graded motor imagery. Such cross-condition inferences are grounded in biological plausibility and are explicitly presented as theoretical hypotheses rather than mechanisms empirically established in PFP.

Furthermore, statements reflecting the authors’ clinical experience or opinion (e.g., “the authors propose,” “in the authors’ clinical experience”) represent the lowest level within the evidence hierarchy and are explicitly marked as such throughout the manuscript to ensure a clear distinction from conclusions derived directly from the reviewed evidence.

## Methods

In accordance with a narrative review design, this section aims to synthesize current evidence on multimodal rehabilitation strategies for peripheral facial palsy. We *a priori* distinguished three domains: (A) facial palsy and related diagnoses, (B) assessment of facial somatosensory distortion and body representation, and (C) interventions targeting facial somatosensory distortion. Studies were eligible when they addressed combined constellations (34).

### Search strategy

We developed predefined search strings and applied them in major biomedical databases (e.g., PubMed, Embase, CINAHL, and Web of Science). *Constellation A* (facial palsy) was operationalized using terms related to facial nerve disorders and their synonyms, including “Facial Nerve Diseases,” “Facial Paralysis,” “Peripheral Facial Palsy,” “Bell’s palsy,” “Facial Paresis,” “Facial Nerve Injury,” and “Facial Synkinesis.” *Constellation B* (assessment of facial somatosensory and body representation) included terms such as “two-point discrimination,” “tactile localization,” “tactile discrimination,” “laterality judgement,” “quantitative sensory testing” (e.g., mechanical and thermal detection thresholds), “tactile shape discrimination,” “texture discrimination,” “facial emotion recognition,” “facial expression recognition,” “self-face recognition,” and “video-based self-confrontation tasks.” *Constellation C* (interventions) included “sensorimotor integration,” “motor learning,” “motor imagery,” “mental practice,” “laterality training,”” left–right training“, “emotion training,” “graded motor imagery,” “mirror therapy,” “action observation,” “multisensory integration,” and “mindfulness-based movement.” Search terms within each constellation were combined with OR, and constellations were combined with AND.

We limited the search to human studies and peer-reviewed journal articles, with no restrictions on study design, and considered publications in English, German, and Dutch from database inception to April 2026. In addition, we screened the reference lists of included articles to identify further relevant studies (35).

### Study selection and data synthesis

Two authors (BT and HP) independently screened titles and abstracts against the predefined A, B, and C domains and subsequently assessed the full texts of potentially eligible articles for conceptual relevance. Disagreements were resolved in consensus meetings. Because of the expected clinical and methodological heterogeneity, we did not perform a formal risk-of-bias assessment or meta-analysis; instead, findings were synthesized narratively with a focus on converging mechanisms and clinically meaningful multimodal rehabilitation strategies (36). Sections derived from Search Strings A + B (Assessment) and A + C (Rehabilitation).

### Facial palsy and assessment (search strategy A + B)

**Figure 2:**
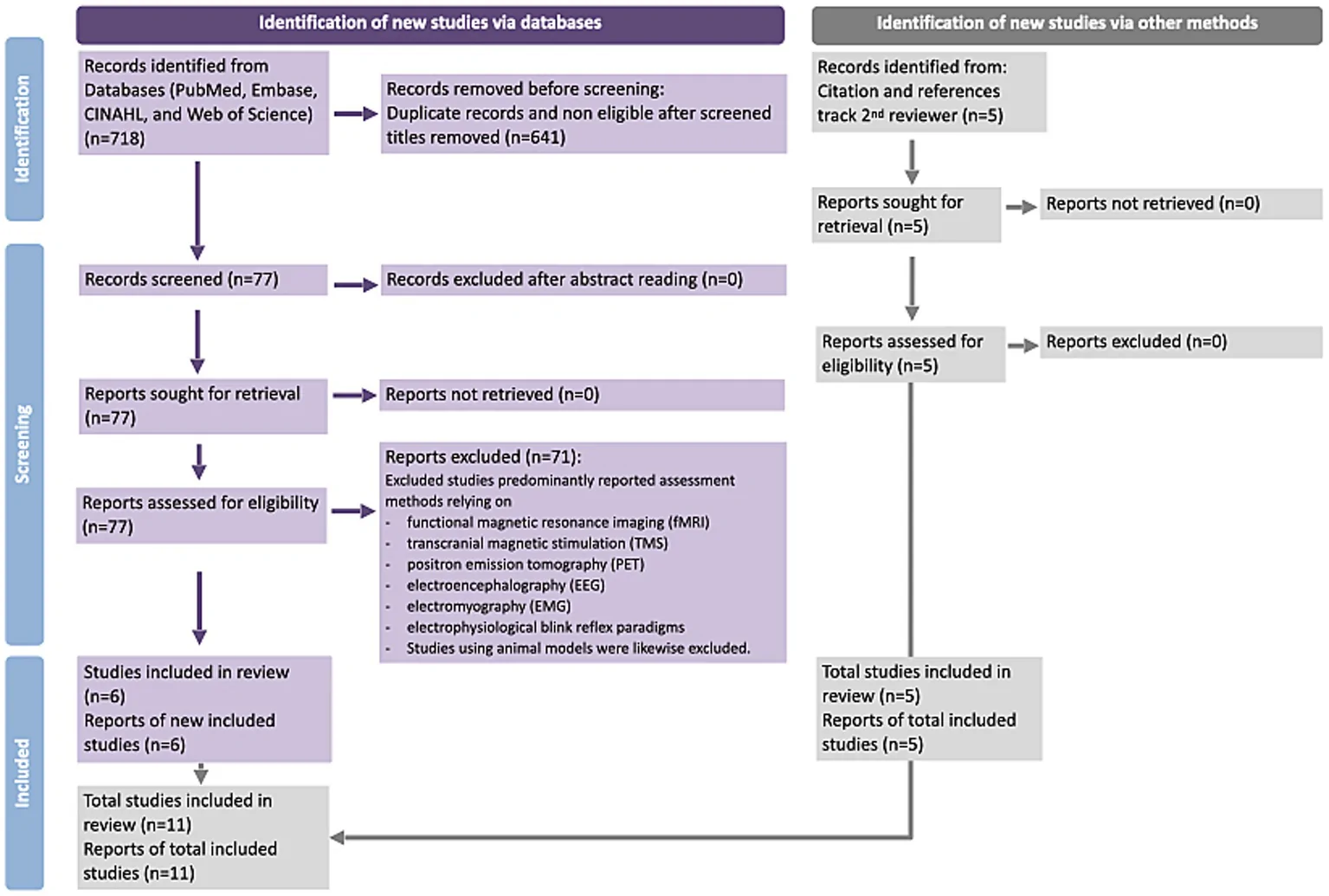
Flow-chart of the search strategy for facial palsy and assessment.

Using the combined search string “peripheral facial paresis” (A) AND “assessments” (B), supplemented by relevant MeSH terms and Boolean search combinations (Search Strategy A + B), a total of 718 records were identified without restrictions regarding publication date, language, or study design. Following removal of duplicate records and screening of titles and abstracts, 77 studies were retained for full-text evaluation. Ultimately, 6 studies (18, 37–41) met the predefined inclusion criteria, as they investigated clinically applicable assessment tools in the context of facial palsy. The 71 excluded studies predominantly reported assessment methods relying on functional magnetic resonance imaging (fMRI) or other neurophysiological techniques, including transcranial magnetic stimulation (TMS), positron emission tomography (PET), electroencephalography (EEG), electromyography (EMG), and electrophysiological blink reflex paradigms. Studies using animal models were likewise excluded. To minimize selection bias, two reviewers independently conducted and cross-validated the screening process. This procedure resulted in the additional inclusion of five studies addressing applied clinical assessments (42–46), yielding a total of 11 studies for the present narrative review which are shortly summarized (Figure 2).

Bate et al. (37) examined facial expression processing in six individuals with Möbius sequence using a standardized neuropsychological battery, including the Ekman 60 Faces Test, the Emotional Hexagon Test, and the Reading the Mind in the Eyes Test, complemented by measures of facial and object imagery and control tasks for face identity and lower-level visual processing (CFPT, CFMT, BORB). These findings may indicate mild-to-moderate impairments in facial emotion recognition, consistent with broader perceptual limitations rather than emotion-specific deficits, thereby providing preliminary evidence against embodied simulation accounts of emotional expression processing.

Bogart (42) investigated socioemotional functioning in individuals with congenital and acquired facial palsy using validated self-report instruments assessing emotional clarity, attachment security, perceived stigma, anxiety, and depression. Both groups demonstrated elevated levels of anxiety, depression, and stigma relative to normative data; however, reduced emotional clarity and insecure attachment were observed exclusively in the acquired facial palsy group, supporting the hypothesis of a congenital socioemotional advantage through adaptive developmental compensation.

Carneiro Pereira and Vannuscorps (43) investigated the role of motor simulation in automatic facial expression recognition by employing a dual-task paradigm in which individuals with congenital facial palsy (Möbius syndrome) categorized emotional sounds while being incidentally exposed to congruent or incongruent facial expressions. Both groups exhibited robust and comparable congruency effects—that is, superior performance in congruent conditions—providing strong evidence that efficient and automatic facial expression recognition can occur independently of motor simulation, challenging simulation-based theories of social cognition. Although Möbius syndrome is not a classical peripheral facial palsy such as Bell’s palsy, we included it in our results because it involves facial nerve dysfunction with bilateral motor impairment and therefore fits our hypothesis regarding a somatosensory and body image/body schema-related approach.

De Carvalho et al. (39) assessed body image in 40 adults with long-standing unilateral facial palsy using the Body Image Quality of Life Inventory (BIQLI) before and at 15 and 180 days following botulinum toxin treatment. Baseline BIQLI scores were markedly negative across all domains. Following treatment, body image improved progressively, with several domains showing positive scores at the 180-day assessment; however, certain areas, including work/school performance and aspects of sexuality, remained negatively affected, indicating that complete body image recovery is not uniformly achieved.

Dring et al. (40) employed a cross-sectional survey design to examine social anxiety in individuals with facial palsy using a multidimensional self-report battery, comprising the Social Appearance Anxiety Scale (SAAS), the SIAS-6/SPS-6 for general social anxiety, the Brief Fear of Negative Evaluation Scale (BFNE), and the Body Image Coping Strategies Inventory—Appearance-Fixing Subscale (BICSI-AF). Depression was assessed with the PHQ-8 and facial function with the Facial Disability Index (FDI-PF). Individuals with facial palsy reported significantly elevated scores across all psychosocial measures compared to controls. Fear of negative evaluation and appearance-fixing behaviors emerged as the primary predictors of social appearance anxiety.

Gil-Martínez et al. (41) used a case series design where the primary outcome measure was surface EMG activity of key facial muscles (*M. orbicularis* oris, M. buccinator, and M frontalis) recorded during standardized facial gestures with and without visual biofeedback, enabling detailed analysis of agonist activation patterns and synkinesis. The Facial Disability Index (FDI) served as a complementary patient-reported outcome, capturing physical and social disability. This multimodal assessment approach provided mechanistic insight into the immediate neuromuscular effects of computerized mirror visual feedback.

Japee et al. (44) assessed facial emotion perception in congenital facial palsy using a multi-layered psychophysical battery of computer-based emotion detection and recognition tasks. Static and dynamic expression tasks based on morph continua and variable emotional intensity were used to derive perceptual threshold measures, supplemented by multiple control tasks targeting facial identity, motion, and body expression perception. A subset of participants underwent fMRI to explore the neural correlates of expression processing. The study revealed selectively elevated facial emotion detection thresholds in facial palsy, with largely preserved non-emotional and body-related perception, and linked these subtle perceptual deficits to altered neural activity in emotion- and face-sensitive brain regions.

Kuttenreich et al. (45) examined facial emotion recognition in acquired, unilateral, chronic peripheral facial palsy with synkinesis and healthy controls, matched for gender, age, and education. An additional measurement of auditory emotion recognition allowed for a distinction between modality-specific and cross-modal performance. Self-assessment was collected using questionnaires. Compared to healthy controls, patients with peripheral facial palsy showed no significant differences in measured facial and auditory emotion recognition or self-assessment. A risk of impairment was identified only in isolated cases with pronounced facial asymmetry and pronounced facial synkinesis.

Nguyen and Coulson (38) adopted a mixed-methods design to assess the psychosocial burden of pregnancy-associated Bell’s palsy. The assessment framework centered on patient-reported outcome measures, including the Facial Disability Index (FDI) for functional and social impairment and the Mirror Gazing Cognition and Affect Rating Scale (MG-CARS) for appearance-related distress and self-perception, supplemented by a custom quality-of-life questionnaire and open-ended survey responses subjected to thematic analysis. This integrative approach provided a multidimensional characterization of the impact of facial palsy on daily functioning, interpersonal relationships, and psychological well-being.

Storbeck et al. (46) investigated emotion recognition longitudinally in acute Bell’s palsy using a computerized multi-task battery, combining multiple psychophysical EMO tasks, including composite face paradigms, dynamic videos with variable emotional intensity and orientation, visual search, and the Diagnostic Analysis of Nonverbal Accuracy (DANVA), with both accuracy and reaction time as primary outcome parameters. A control FACE task was included to disambiguate emotion-specific processing from general face perception. Complementary assessments comprised clinician-rated facial function (FNGS 2.0) and patient-reported measures (FaCE, HADS, SF-12). Facial emotion recognition accuracy was preserved; however, reaction times were significantly prolonged, indicating delayed rather than impaired processing. This shows a pattern consistent with disrupted embodied simulation.

Volk et al. (18) applied the DFNS Quantitative Sensory Testing (QST) protocol to characterize somatosensory function at the paretic side, contralateral side, and a distal control site in patients with acute and chronic facial palsy, expressing all 13 parameters as z-scores against age- and sex-adjusted normative data. Patient-reported outcomes included the FaCE facial comfort subscale and the SF-36 pain domain. Somatosensory function was largely preserved across all QST parameters, with no clinically relevant asymmetry between affected and unaffected sides, despite patients reporting marked subjective sensory complaints. This dissociation supports the idea of a cortical mismatch hypothesis rather than peripheral sensory pathology.

Supplementary Table 1 provides an overview of the discussed studies including main categories of assessments, study design and level of evidence.

### Conclusion: assessment studies

Eleven studies were identified that investigated clinically applicable assessment approaches beyond neuroimaging and neurophysiological techniques such as fMRI, EEG, or TMS, thereby offering greater translational potential for clinical practice. One study specifically focused on somatosensory profiling using QST (18); the majority of the remaining studies addressed facial and/or emotional expression recognition and auditory emotion recognition (37, 43–46) or socioemotional functioning and psychological processing (40, 42). Facial function and motor performance were incorporated as secondary outcomes in several studies (38, 40, 41), whereas alterations in orofacial body image were explicitly examined in only two studies (39, 40). It should be noted that several of the reviewed studies were conducted in individuals with congenital facial palsy (e.g., Möbius sequence), whose lifelong absence of facial motor and sensory input likely engages fundamentally different adaptive and compensatory mechanisms than those following acquired nerve injury in adulthood. Findings from these populations should therefore be interpreted as hypothesis-generating rather than directly transferable to patients with acquired peripheral facial palsy.

Notably, none of the identified studies constitutes a large-scale, high-quality randomized controlled trial (RCT). Rather, the included literature comprises small cohort studies, case–control designs, cross-sectional surveys, and service evaluations, predominantly situated at the interface of physiotherapy, rehabilitation medicine, and psychology.

The overall level of evidence ranges from low to moderate, which reflects the nature of the research questions and the available study designs. One study achieved Level II–III evidence (46), incorporating a control group and repeated assessments; however, even this study was limited by a small sample size despite a reported power calculation. Eight studies were rated Level III, as they employed observational cohort or cross-sectional designs (18, 38–40, 42–45), and two studies were assigned Level IV, as they reported case series without control groups and with very small, uncontrolled samples (37, 41). Overall, the evidence base for reliable, clinically applicable assessment tools in peripheral facial palsy remains limited; however, the identified instruments may collectively hold potential for integration into a structured, multimodal assessment pathway. Such a pathway could support healthcare professionals, including specialized physiotherapists and speech and language therapists, in delivering systematic, person-centered management of this clinically and psychosocially complex condition.

### Facial palsy and rehabilitation (search strategy A + C)

**Figure 3:**
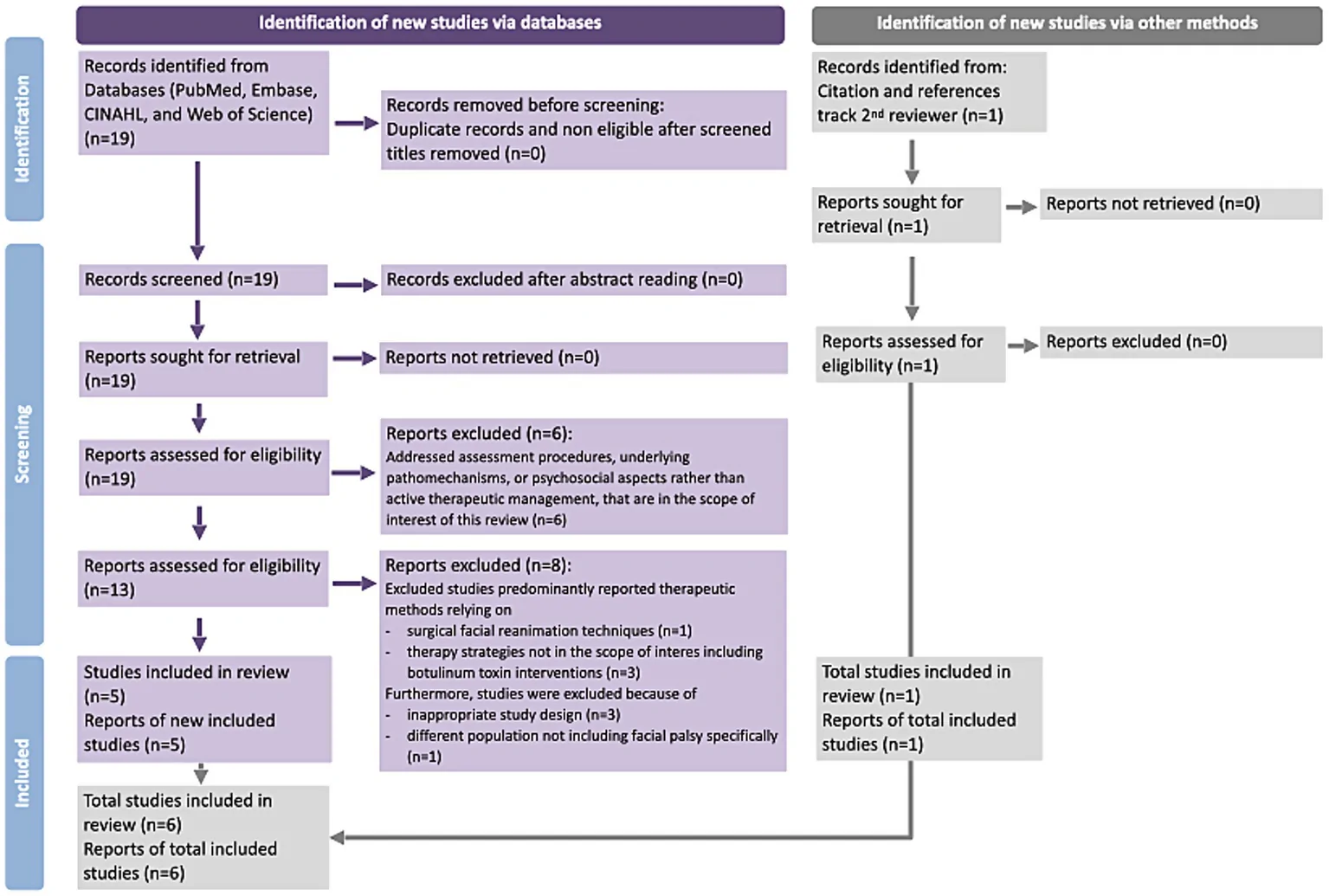
Flow-chart of the search strategy for facial palsy and rehabilitation.

Using the search string “peripheral facial paresis” AND “rehabilitation” (Search Strategy A + C), a total of 19 articles published up till April 2026 were identified. Following full-text screening, 13 studies were classified as explicitly therapy-oriented rehabilitation papers, whereas 6 articles (18, 42, 44, 46–48) primarily addressed assessment procedures, underlying pathomechanisms, or psychosocial aspects rather than active therapeutic management, that are in the scope of interest of this review. Of the 13 rehabilitation-focused studies, one article (49) dealt exclusively with surgical facial reanimation techniques, including neurotization and muscle transfer procedures, and was therefore excluded from the present review, which is restricted to conservative, non-surgical rehabilitation approaches. Seven more studies had to be excluded because of therapy strategies that were not in the scope of interest of this review including botulinum toxin interventions (50–52), an inappropriate study design (53–55) and a different population not including facial palsy specifically (56) resulting in five final studies included. To minimize selection bias, two reviewers independently conducted and cross-validated the screening process. This procedure resulted in the additional inclusion of one study addressing the rehabilitation strategies of interest (57). Accordingly, six studies (41, 57–61) fulfilled the inclusion criteria for the rehabilitation section of this review which is summarized below (Figure 3).

Barth et al. (58) demonstrated that the addition of Mirror Book Therapy (MBT) to standard neuromuscular facial rehabilitation in idiopathic facial palsy yielded significantly greater improvements in facial function. Outcome was measured by the Facial Grading Scale (FGS), the Facial Disability Index Physical (FDIP), and the Facial Disability Index Social (FDIS). The intervention was compared with standard care alone, with the most pronounced effect observed in the domain of social participation. These findings suggest that sustained exposure to a symmetrical facial image can favorably modulate both cortical motor output and the patients´ self-perception of facial appearance.

Gil-Martínez et al. (41) extended this theoretical framework by employing computerized mirror visual feedback: their Specular Face software delivered a real-time symmetrical facial image while surface EMG simultaneously recorded muscular activity. In this single-session case series, immediate reductions in synkinetic co-activation and improvements in agonist activation were observed in 4 of 5 participants, providing mechanistic support for the concept of visually mediated “brain training” in facial palsy rehabilitation. The absence of longitudinal outcome data nonetheless limits the interpretation of sustained treatment efficacy.

Kasahara et al. (61) demonstrated, in an earlier controlled pilot study, that a four-week Tape Feedback Therapy protocol, employing perioral taping as augmented somatosensory biofeedback during eye-closure exercises, reduced oro-ocular synkinesis more effectively than conventional therapy alone. Furthermore taping was more sensitive than finger-based feedback. These findings support the premise that enhanced tactile input can facilitate adaptive cortical re-learning of facial movement patterns and synkinesis suppression.

Bernd et al. (59) introduced a newly developed digital biofeedback program that amplifies subtle facial movements to guide intensive neuromuscular facial training. Software-derived movement scores demonstrated strong correspondence with the House–Brackmann Grading System (HBGS) grading, providing a validated technological platform for the objective measurement and guidance of facial rehabilitation. The study simultaneously documented substantial quality-of-life reductions across all patients (HBGS grades II–IV), highlighting the breadth of functional impairment that rehabilitation programs must address.

Hotton et al. (60) targeted the psychosocial and cognitive dimensions of facial palsy rehabilitation by evaluating six condition-specific self-help guides grounded in Cognitive Behavioral Therapy (CBT), Acceptance and Commitment Therapy (ACT), and social skills training. This pre–post quasi-experimental study demonstrated significant improvements in social functioning and appearance-related distress, even in the absence of measurable change in facial motor function or depression scores (HADS), indicating that targeted psychological rehabilitation can achieve meaningful patient-reported gains independently of peripheral motor recovery.

Zhao et al. (57) evaluated the additive effect of mirror therapy on top of facial proprioceptive neuromuscular facilitation (PNF) and standard medical care in acute peripheral facial palsy within a single-centre randomized controlled trial. In 34 patients with symptom duration ≤7 days, combining mirror therapy with pharmacotherapy (including early high-dose dexamethasone), acupuncture, electrostimulation and ultrashort-wave therapy led to significantly higher FaCE scores, a greater proportion of patients achieving House–Brackmann grade I, and a substantially shorter clinical recovery time compared with conventional care plus PNF alone. The intervention was well tolerated, with no adverse events reported, suggesting that integrating mirror therapy into early facial PNF-based rehabilitation may meaningfully accelerate functional recovery in acute peripheral facial palsy.

Supplementary Table 2 provides an overview of the discussed studies including main categories of rehabilitation, study design and level of evidence.

### Conclusion: rehabilitation studies (A + C)

Overall, only six non-surgical studies on conservative rehabilitation for peripheral facial palsy were identified that explicitly targeted craniofacial body-distortion, encompassing disturbances in somatosensory body schema, visual body image, and self-related appearance evaluation. All the reviewed studies examined therapy-based interventions. Four of the studies focused on neuromuscular or biofeedback-based approaches for facial training (41, 57, 58, 61). These approaches included mirror therapy combined with PNF (proprioceptive neuromuscular facilitation), computer-guided visual mirror feedback combined with electromyography (EMG), and somatosensory feedback via tape. One study (59) examined the validity of a digital biofeedback platform designed to guide facial exercises. Another study (60) evaluated structured psychological self-help interventions intended to supplement rehabilitation. Together, these studies provide preliminary, converging evidence suggesting that an integrated approach combining visual and somatosensory feedback, structured neuromuscular training, and targeted psychosocial support may be necessary for effective rehabilitation of peripheral facial palsy. However, none of the included studies are large-scale, high-quality randomized controlled trials. Instead, the current body of evidence is limited to small-scale pilot studies and service evaluations at the intersection of physical therapy, rehabilitation medicine, and psychology.

In terms of evidence level, the overall strength ranges from low to moderate, with one notable exception. Zhao et al. (57) achieved a Level II rating by conducting a randomized controlled trial (RCT). Kasahara et al. (61) received a Level II–III rating for a small, controlled pilot study. Barth et al. (58) and Hotton et al. (60) received a Level III rating for non-randomized comparative or pre-post studies. Gil-Martínez et al. (41) and Bernd et al. (59) received a Level III–IV rating for uncontrolled case series and pilot validation studies, respectively. Despite their conceptual relevance to conservative rehabilitation, these findings reflect a strikingly limited empirical basis. Sample sizes are consistently small, study designs are predominantly non-randomized, follow-up durations are short, and long-term functional outcomes are rarely reported. Consequently, the current evidence only preliminarily supports conservative, task-oriented interventions for peripheral facial palsy, such as mirror therapy, biofeedback techniques, and targeted psychosocial rehabilitation. Furthermore, the authors argue that there is a clear and urgent need for well-designed, sufficiently powered randomized controlled trials to inform evidence-based clinical guidelines in favor of innovative, systematic training programs that more intensively target brain networks.

### Clinical implications for further assessment, intervention and research

This review article highlights a significant gap in the evidence regarding both the assessment and non-surgical rehabilitation of peripheral facial palsy, with a focus on craniofacial somatosensory disruption. Despite a well-established theoretical understanding of cortical reorganization, somatosensory mismatch, and a disrupted orofacial body schema, as described in the literature review, the available evidence regarding the management of craniofacial somatosensory distortion remains scarce. This empirical gap stands in strong contrast to the well-documented functional, emotional, and psychosocial burden that peripheral facial palsy imposes on affected individuals, a burden that persists regardless of the objective severity of the motor disorder and that still receives far too little attention in current clinical practice (62, 63).

Against this background, two interrelated priorities for clinical assessment and research emerge to improve clinical practice in peripheral facial palsy. First, it is necessary to establish a standardized, multimodal assessment protocol that allows for the systematic mapping of disturbances in the orofacial body schema and associated somatosensory changes. Second, the development of a systematic approach to brain training is proposed as a necessary step toward evidence-based, centrally focused rehabilitation. Both priorities are elaborated upon in the following sections.

## Assessment

Useful assessments in the management of facial palsy can be divided into screening questionnaires or patient reported outcome measures (PROMS) and standardized clinical tests.

### Questionnaires

#### Established assessments

Before introducing the proposed screening tools for craniofacial somatosensory distortion (CFSD), it is important to acknowledge two established grading systems that provide a general clinical impression of facial palsy. Another test, that should be mentioned as well is the Facial Disability Index (FDI).

*The House-Brackmann Grading System (HBGS)*, the most widely used clinician-rated scale, offers a six-point global assessment of facial nerve function ranging from normal (Grade I) to total paralysis (Grade VI) (64). *The Sunnybrook Facial Grading System* (*FGS*) provides a more sensitive, quantitative score across three domains: resting symmetry, voluntary movement, and synkinesis. It yields a composite score ranging from 0 to 100 (65). Its reliability makes it well suited for multidisciplinary assessment and treatment (66).

*The Facial Disability Index (FDI)* is a disease-specific, patient-reported outcome measure designed to assess functional disability and psychosocial impact in individuals with disorders of the facial motor system. It consists of 10 items forming two subscales, Physical Function and Social/Well-being, each scored from 0 to 100, with higher scores indicating better function. The FDI has demonstrated good internal consistency, test–retest reliability and construct validity in patients with peripheral facial palsy and has been translated and validated in multiple languages, making it a suitable tool to complement clinician-rated facial grading systems in both clinical practice and research (67–69).

While the HBGS and FGS capture motor function, symmetry, and synkinesis, and the FDI includes psychosocial wellbeing, neither addresses somatosensory, perceptual, nor body-schema disturbances, which are increasingly recognized as CFSD. Based on the reviewed literature and the authors’ clinical experience, we propose four questionnaires and screening tools to assess deficits, signs, and symptoms suggestive of CFSD in patients with facial palsy. These tools may guide subsequent clinical assessment and rehabilitation strategies.

#### Assessment focused on (facial) body distortion

Assessments that focus more on the emphasis of this article like body image, motor expression and body awareness are the Mirror Gazing Cognition and Affect Rating Scale (MG-CARS), the Body Image Quality of Life Inventory (BIQLI), the Fremantle Body Awareness Questionnaire (face), and the Tongue and Mouth Imagery Questionnaire (TMIQ).

*The Mirror Gazing Cognition and Affect Rating Scale (MG-CARS)* is an experimental rating instrument developed in the context of mirror-gazing paradigms for body dysmorphic disorder. It assesses appearance-related cognitions (e.g., perceived defectiveness, dissatisfaction with specific facial features) and affective responses such as distress, anxiety, sadness and anger immediately following a standardized mirror exposure. In facial palsy, MG-CARS might be used as a brief patient-reported outcome to capture cognitive–affective reactions to viewing one’s own face, thereby complementing objective facial grading measures. Although formal psychometric properties (e.g., test–retest reliability, internal consistency) have not been reported in a dedicated validation study, repeated use in experimental mirror-gazing research suggests that MG-CARS is sensitive to between-group differences and to changes in appearance-related distress following experimental manipulations (70, 71).

The *Body Image Quality of Life Inventory (BIQLI)* assesses the impact of body image on different domains of daily life and has been used to evaluate changes in body-image-related quality of life in patients with facial palsy undergoing botulinum toxin treatment, demonstrating sensitivity to clinically meaningful improvements in this population (39).

*The Fremantle Body Awareness Questionnaire (face)* is a region-specific adaptation of the Fremantle Awareness Questionnaire family, designed to assess distorted self-perception and body image of the face. It consists of a brief set of items rated on a 5-point Likert scale (0 = never to 4 = always), with higher scores indicating greater disturbance of facial body awareness (e.g., feelings that the face looks or feels different, foreign or difficult to control). Fremantle questionnaires for other body regions (e.g., back, knee, neck) have demonstrated acceptable internal consistency, test–retest reliability and construct validity in individuals with and without pain, supporting their use as patient-reported outcomes to quantify altered body perception. In the context of facial palsy, the face-specific version can therefore be used as an exploratory tool to capture changes in perceived facial self-image and body awareness alongside clinician-rated facial function measures (72, 73).

*The Tongue and Mouth Imagery Questionnaire (TMIQ)* is a newly developed instrument to assess motor imagery (MI) in the orofacial region. It comprises two subscales, kinesthetic MI (kMI) and visual MI (vMI), which together yield a total motor imagery score. The questionnaire includes five movement items: patients first perform each movement, then imagine it from a first-person perspective, rating the clarity of the visual image (vMI) and the intensity of the imagined movement (kMI) on 5-point scales, as well as the pain intensity during execution or imagery on a 0–10 scale. Subscale scores range from 5 to 25 for kMI and vMI, 10–50 for the total MI score, and 0–50 for pain, with higher MI scores indicating clearer or more intense imagery and higher pain scores indicating greater pain; the TMIQ has demonstrated good reliability and validity in patients with temporomandibular disorders and healthy controls (74).

### Clinical tests

Next, to the established clinical testing in peripheral facial palsy like assessing oral and facial function, neuromusculoskeletal screening of craniofacial, cranio-cervical or temporomandibular regions and a proper neurological bed side testing (75, 76) we especially want to emphasis again on clinical procedures that focus more on body awareness, body image and / or schema and somatosensory functions, as these domains are increasingly recognized as relevant components in neurological rehabilitation and facial palsy assessment (18, 26).

**Figure 4:**
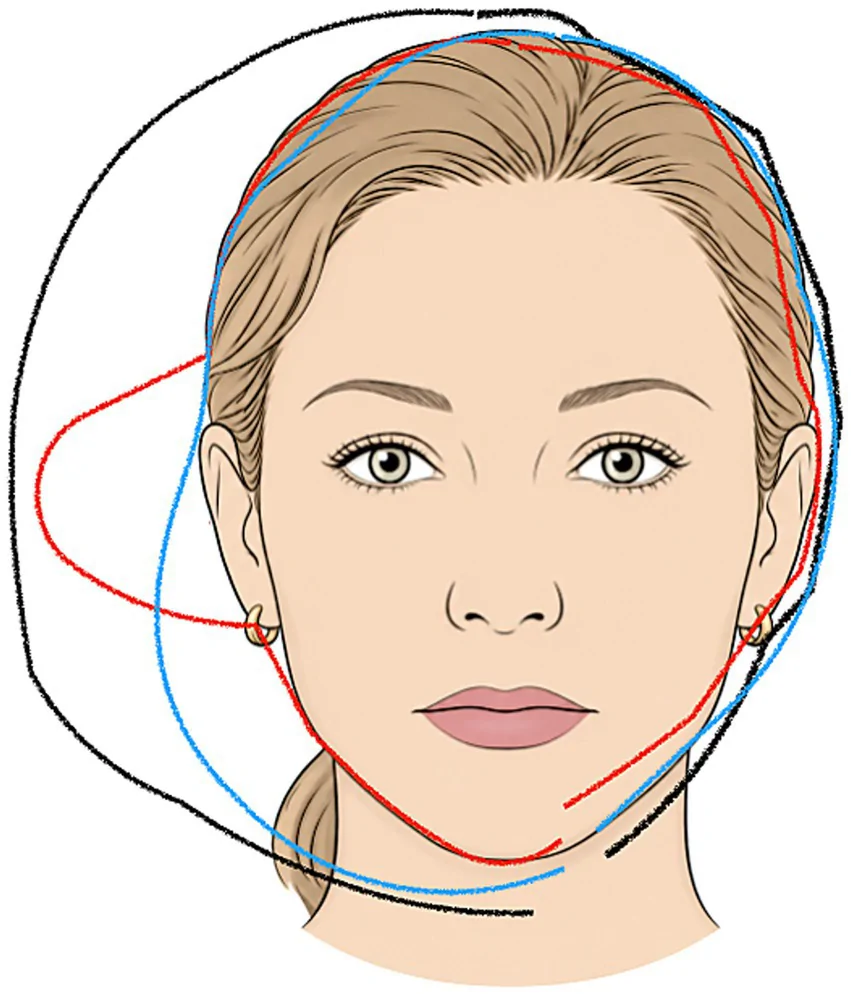
A facial body chart illustrating altered facial perception in a patient with right-sided Bell’s palsy, tinnitus, and otalgia. The patient independently completed the body chart. The black contour depicts perceived facial boundaries; the red contour, pain and discomfort; and the blue contour, perceived tension and reduced sense of control. The pronounced asymmetry between the affected and unaffected sides suggests disturbances in facial body representation and sensorimotor integration.

*Facial body scanning (FBS).* The facial body scan test consists of a verbal or audio recording in which patients must systematically focus on different areas of the face, such as the eyes, ears, mouth, tongue, and teeth and different qualities like size, temperature, left/right comparison, (dis)comfort, tension vs. relaxation. The patients’ individual perceptions are recorded together with the therapists in a body scan protocol and a body schema table (Figure 4). Good validity of the pain drawings, with acceptable sensitivity and specificity and a high level of interrater agreement are indicated (77, 78). To complete the FBS, participants use colored pencils whose color and shape jointly encode the and intensity (0–3) of their perceived sensations, following a pain-drawing methodology that has shown high test–retest reliability in patients with temporomandibular disorders (79). This type of test is also used to assess body awareness of the orofacial region in woodwind and brass instrumentalists (80).

**Figure 5:**
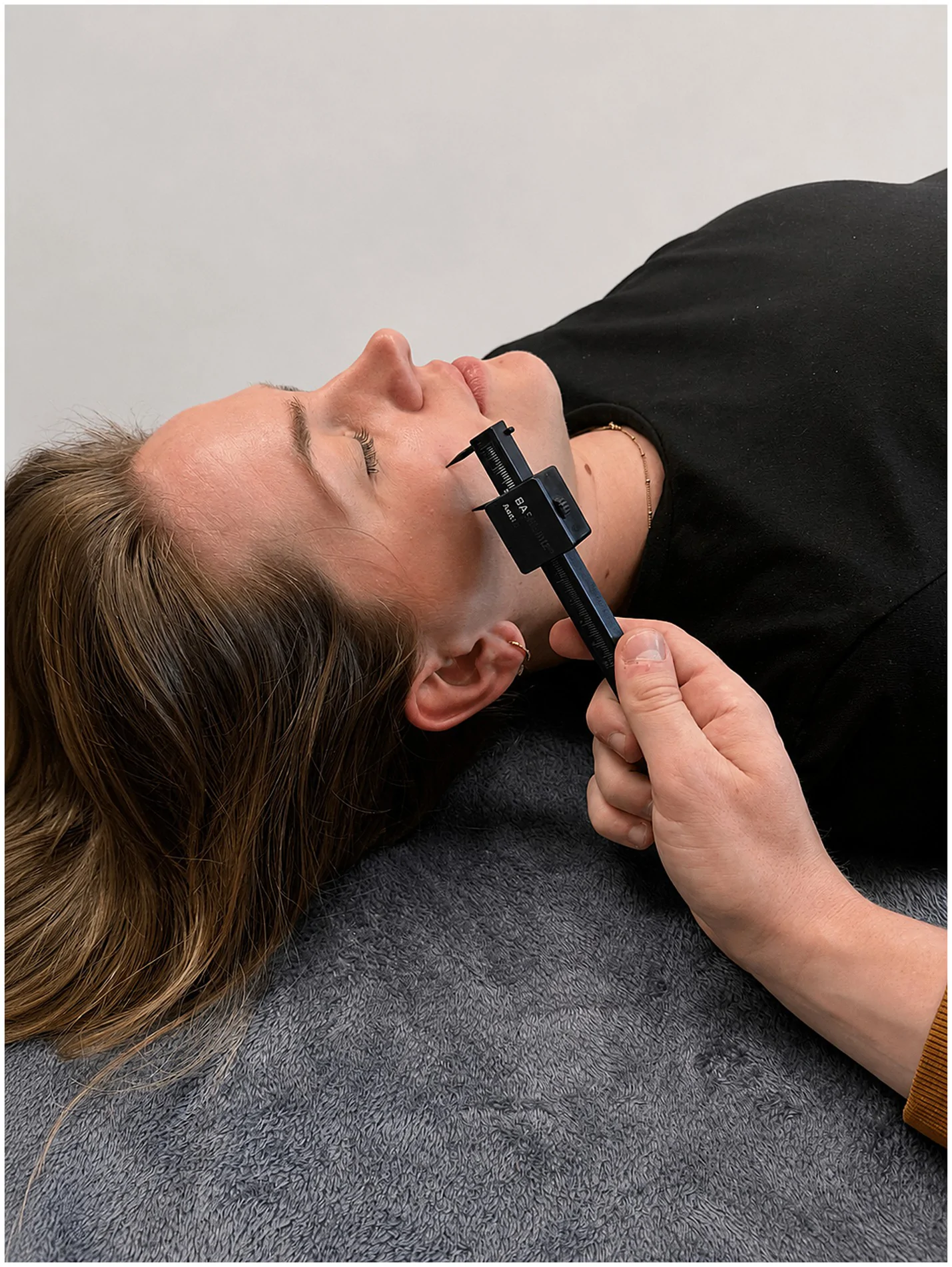
Two-point discrimination (TPD) assessment in the trigeminal region. Written informed consent was obtained from the individual(s) for the publication of these identifiable images in this article.

*Two-point discrimination test (TPD test).* The well-established *two-point discrimination test (TPD test)* is a method for assessing tactile sensitivity and sensory perception, particularly in the trigeminal region (Figure 5). It is frequently used to measure the smallest spatially perceived distance on the skin, that is, the ability to perceive two closely spaced touch points as separate stimuli by using a special instrument (baseline tool) consisting of a plastic wheel with prongs. These prongs are spaced at specific intervals, starting at 25 mm, then 20 mm, and finally decreasing by 1 mm from 15 mm down to 2 mm (81).

**Figure 6:**
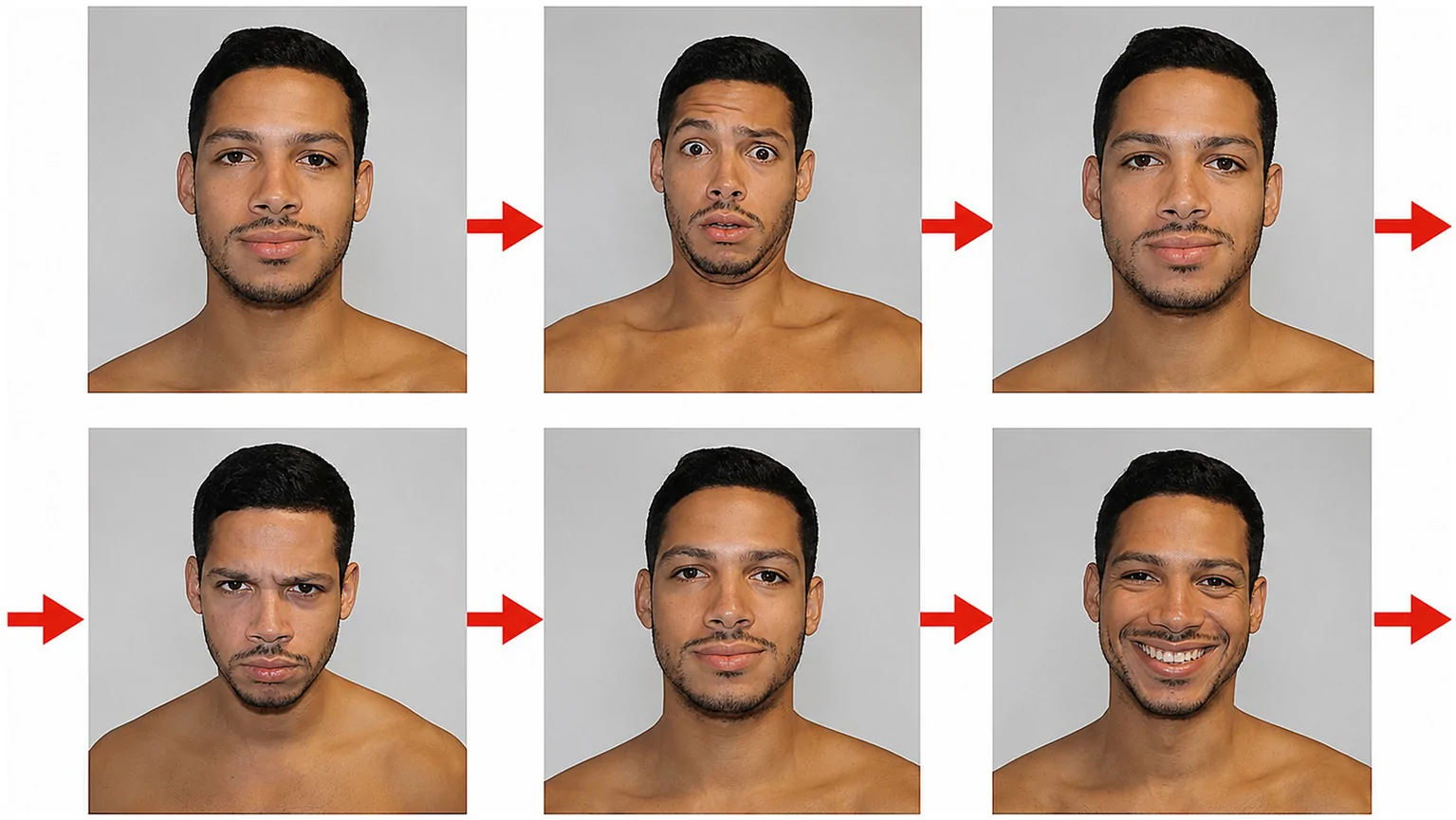
Facial emotion recognition. A standardized assessment consisting of 49 photographs from seven different models, in which a neutral facial expression changes into a basic emotion within 0.5 s. As illustrated, the sequence may progress from neutral → surprise → neutral → anger → neutral → happiness. The system records emotion recognition accuracy, performance during transitions between emotions, and response speed, providing a comprehensive evaluation of an individual’s emotion recognition abilities (https://www.myfacetraining.com). Written informed consent was obtained from the individual(s) for the publication of these identifiable images in this article.

*Facial Emotion Recognition (FER) and Lateralization (LAT)* can both be tested using the “My Face Training” software.^1^ This software provides a standardized method for assessing the ability to recognize emotions based on facial expressions and to identify differences in facial expressions on the left or right side of the face (Figure 6). Both tests are particularly relevant in the clinical examination of patients with facial pain, as studies show that affected individuals tend to perform worse on these tests than healthy control subjects (82).

*Trigeminal Blink Reflex Test (TBRT).* The trigeminal blink reflex was elicited using a standardized pendulum-ball paradigm. In this paradigm, a controlled mechanical stimulus approaches the face and triggers a reflexive eye-closing movement. This movement is mediated by brainstem circuits (83).

**Figure 7:**
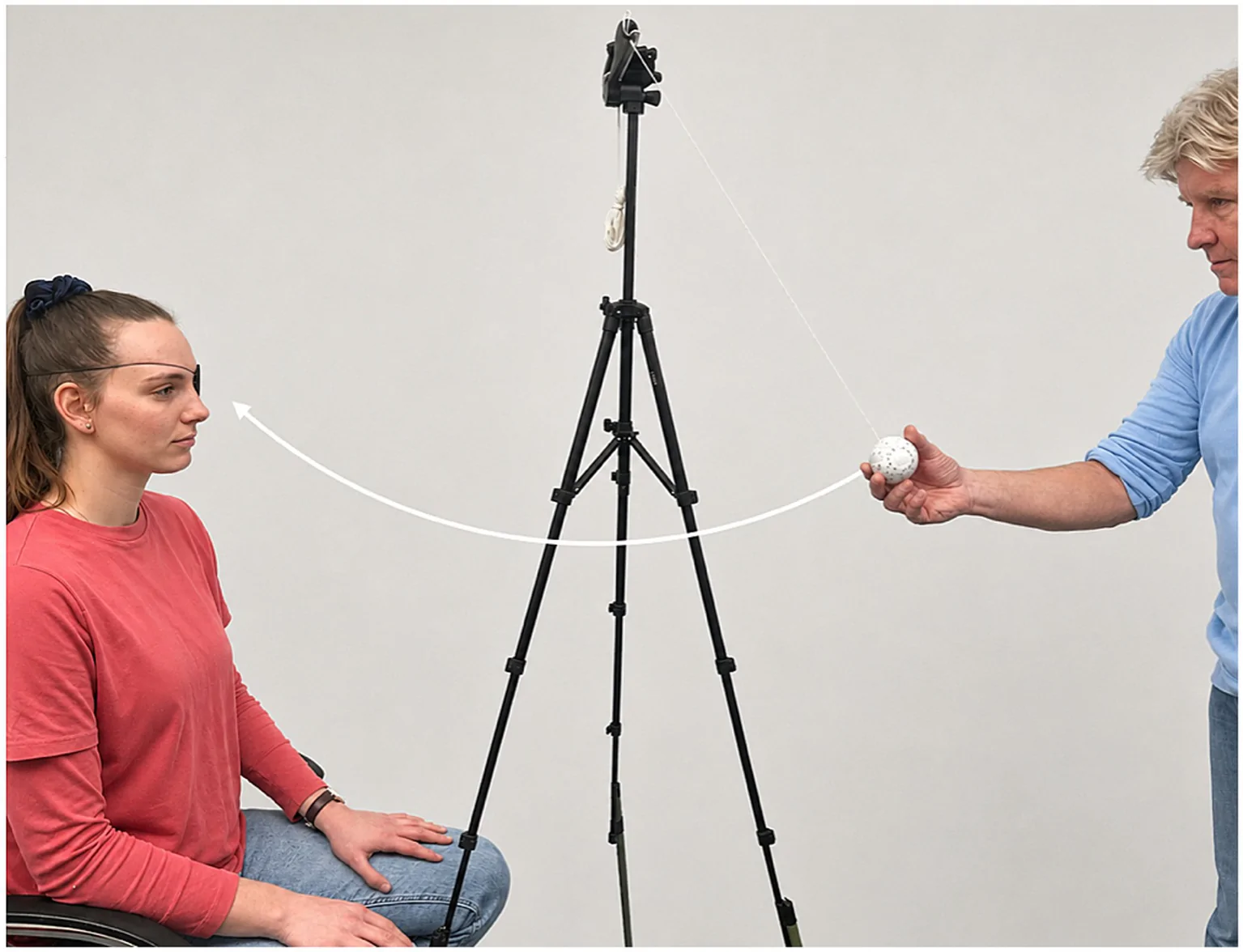
Trigeminal blink reflex (TBR) assessment. A pendulum-mounted ball is swung toward the participant’s eye while a camera records the blink response. The distance between the ball and the glabella at the moment of reflexive eye closure is used as the primary outcome measure. Written informed consent was obtained from the individual(s) for the publication of these identifiable images in this article.

A ball with a diameter of 7 cm is suspended from a pendulum frame attached to the floor and swung in a controlled trajectory toward the patient’s eye. The length of the cord is adjusted so that the ball passed 1–2 cm in front of the nose. Patients sit in a fixed, marked position and are instructed to remain calm and look straight ahead. A laterally positioned, calibrated camera recorded the distance (mm) between the glabella and the ball at the moment of reflexive eye closure. This distance served as the primary outcome measure. Previous studies have demonstrated the trigeminal blink reflex’s excellent internal consistency (Cronbach’s *α* = 0.98), supporting its reliability as a primary outcome measure (84). Although the TBR is highly standardized, it remains sensitive to factors such as attention, anxiety, and expectation (Figure 7).

*Hearing Acuity Test (HAT)*. The facial nerve innervates the stapedius muscle; therefore, peripheral facial palsy often causes ipsilateral hyperacusis and altered loudness perception. This demonstrates that auditory processing is directly involved in the clinical presentation (85). Furthermore, the face serves as the primary reference point for defensive peripersonal space (DPPS), a multimodal representation where tactile, visual, and auditory cues from the near environment are integrated within brainstem and parietal-premotor circuits. Thus, a hearing acuity test is a clinically meaningful way to evaluate the auditory aspect of craniofacial somatosensory distortion in facial palsy (83).

Auditory acuity is measured to detect differences in auditory perception that might indicate changes in the auditory component of the DPPS. An Android/iOS app with variable frequencies and sound intensities can be used to assess auditory discrimination in terms of localization, distance, frequency, and intensity (86). Currently, there are no normative reference values available.

A laser rangefinder is attached to mobile telephone running the Tone Generator app. Two low frequencies (200 Hz and 50 Hz) are selected to investigate potential changes in depth perception at low frequencies because standard audiometry typically uses higher frequencies (250–8,000 Hz) (87, 88).

**Figure 8:**
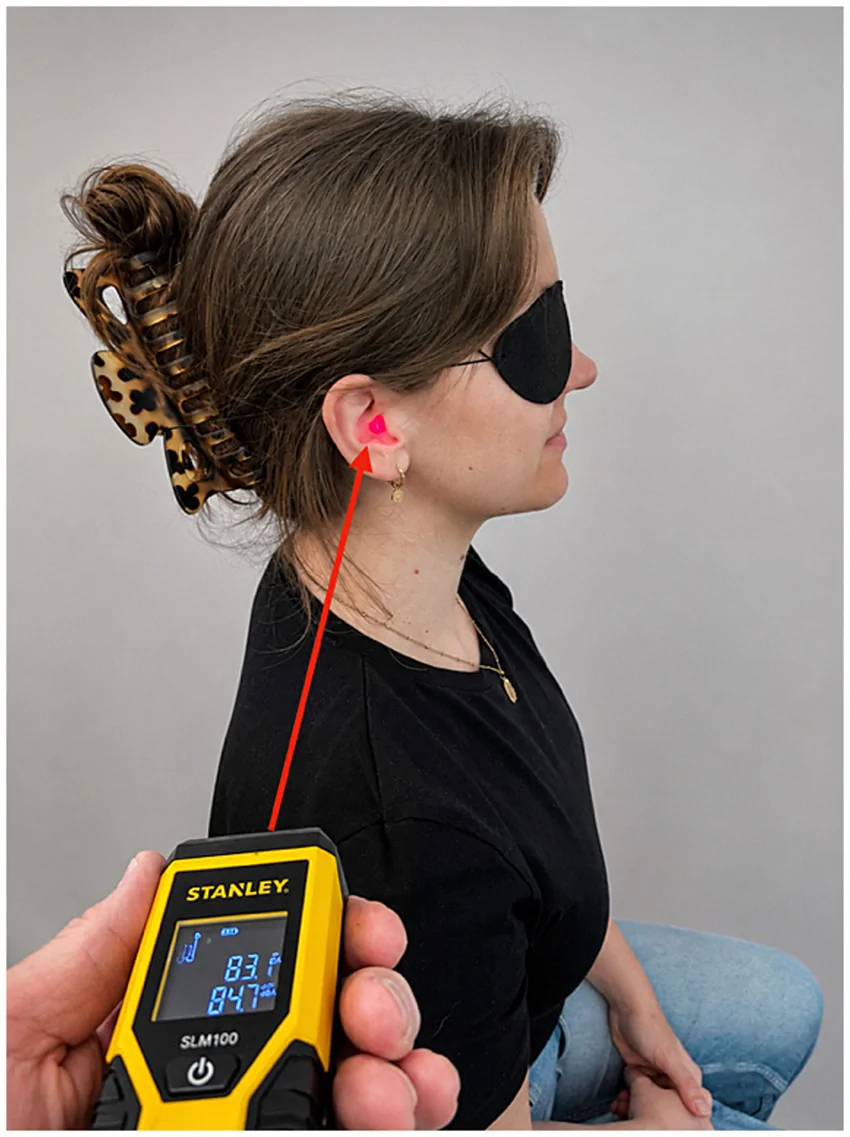
Hearing acuity test (HAT). Low-frequency auditory stimuli (50 Hz and 200 Hz) are presented through a mobile application while the sound source is gradually moved away from the participant. The distance at which a change in the acoustic stimulus is first perceived is recorded as an indicator of auditory sensory sensitivity.

The patient sits in a closed, quiet room with an eye patch covering the eye on the tested side; the sound is played through the mobile phones built-in speakers. The patient is instructed to report any perceived change in the acoustic stimulus. The clinician moves away 20 cm from the starting position, and the distance at which the patient first reported perception is recorded using a laser distance meter. First, both sides are tested at 50 Hz, and then at 200 Hz, since the latter frequency often produces an unpleasant high tone (Figure 8). Although subjective and environmental factors remain a potential source of bias, measuring the hearing threshold via an app has demonstrated good reliability (Cronbach’s *α* = 0.92; ICC = 0.85) (87). This variable can also be incorporated into the arsenal of interventions.

This summary outlines how traditional clinician-rated assessment techniques such as the House-Brackmann scale and the Sunnybrook Facial Grading System are complemented by mental facial scanning with pain drawings, two-point discrimination, facial emotion recognition and lateralization tasks, trigeminal blink reflex testing, and low-frequency hearing acuity assessment, in order to probe defensive peripersonal space and altered facial self-representation. Together, these tools are intended to screen, quantify, and monitor craniofacial somatosensory disturbances and to support individualized, brain-oriented rehabilitation strategies in patients with facial palsy.

## Intervention

After the rationale for the intervention is outlined, the 8-E model is introduced as a clinical guide that provides a structured, neurocognitive framework for rehabilitating peripheral facial palsy, with a particular focus on craniofacial somatosensory distortion. Systematic somatosensory training techniques, such as two-point discrimination training, auditory acuity training, and trigeminal blink reflex training, can be used to provide controlled variations in somatosensory input. These variations can be used to challenge and enhance sensory discrimination, sensorimotor integration, and cortical representation. It is important to note that the 8-E model is presented as a hypothesis-generating conceptual framework rather than a clinically validated rehabilitation protocol. While its individual components (e.g., motor imagery, laterality training, mirror therapy, emotion recognition, action observation, graded exposure, mindfulness, and manual therapy) have each been investigated to varying degrees, their combined application within this model has not yet been evaluated in patients with peripheral facial palsy.

### Challenging of maladaptive cortical reorganization

Following damage to the facial nerve, the motor cortex undergoes rapid and long-lasting reorganization. Studies using transcranial magnetic stimulation (TMS) to map the motor cortex reveal that representations on the affected side increase in size and that additional cortical areas become active (19). Furthermore, functional magnetic resonance imaging (fMRI) studies at rest and during tasks show that patients with synkinesis exhibit greater bilateral activity during voluntary facial movements compared to healthy subjects. This indicates abnormal simultaneous activation of motor programs selectively engaged during normal facial movements (14). Importantly, these cortical changes are not merely epiphenomenal; their extent correlates with the severity of synkinesis and self-reported disability scores (89). Therefore, systematic brain training that targets the level of motor cortex representation through gradual exposure to motor imagery, laterality assessments, and action observation can constitute a theoretically well-founded intervention strategy.

### Neurocognitive and affective consequences of peripheral facial palsy

Peripheral facial palsy disrupts both the body schema and body image: the loss of voluntary facial movements alters the proprioceptive and efferent input from the facial muscles, leading to a deterioration in predictive sensorimotor models, while the visible facial asymmetry results in a poorer quality of life regarding body image and increased social appearance anxiety (BIQLI, SAAS, FACE-Q Paralysis) (39, 40, 90, 91). In some patients, these disorders reach clinically significant levels corresponding to symptoms of body dysmorphia, including excessive preoccupation with asymmetry, avoidance of mirrors, and compensatory safety-seeking behavior (40, 92).

From the perspective of embodied simulation, reduced facial motor capacity impedes the internal simulation of emotional expressions, leading to delayed recognition of others` emotional facial expressions, especially expressions that depend on periorbital and zygomatic activation, and contributes to social withdrawal and a reduced quality of life (46, 93). At the same time, the dense facial innervation and the large orofacial cortical representation ensure high somatosensory resolution and a significant capacity for experience-dependent neuroplastic change, especially when somatosensory, visual, and affective inputs are combined in structured training paradigms (94–96). Multisensory facial exercises that integrate tactile and visual feedback may help reduce synkinetic patterns, while the activation of emotional motor programs via facial feedback activates limbic networks. This supports a more spontaneous and authentic emotional expression and provides a strong rationale for a systematic brain training framework in peripheral facial palsy (97, 98).

The proposed 8-E model may offer a structured, neurocognitively grounded framework based on the current evidence and the authors´ clinical conviction to implement this brain training approach for peripheral facial palsy in practice. It is based on the mechanisms described above regarding body schema, body image, multisensory integration, and embodied emotion recognition, and guides clinicians through successive phases that emphasize exploration, explicit attention, enriched sensory input, and emotionally meaningful expression. By systematically varying the somatosensory, visual, and affective demands in these steps, the 8-E model aims to optimize experience-dependent plasticity within orofacial sensorimotor and socio-emotional networks. In clinical practice, it thus serves as a pragmatic roadmap for designing individualized interventions that simultaneously target motor control, somatosensory representation, and psychosocial reintegration.

### A systematic brain training framework for peripheral facial palsy

Systematic Brain Training (SBT) can be described as a series of rehabilitation strategies aimed at influencing central neural processing and the cortical representation of the face through targeted sensory, motor, and cognitive exercises, with the goal of improving sensorimotor integration and functional recovery (99). In the context of peripheral facial palsy (PFP), SBT integrates the principles of Graded Motor Imagery (GMI) with the addition of training in recognizing facial emotions and facial expressions. Before discussing the different training levels, it is important to note that, if clinically indicated, subtle orofacial manual therapy and neuroscience education can be incorporated as complementary components in the rehabilitation process.

Conceptually, the program is derived from the GMI framework described by Moseley et al. (100), originally developed and validated in patients with chronic pain and complex regional pain syndrome, and was further operationalized for the rehabilitation of head and facial disorders by von Piekartz and Paris-Alemany (2021) (86). In the current context, this framework has been specifically adapted to PFP, with four primary rehabilitation goals:

1. restoration of voluntary facial muscle function;
2. reduction of synkinesis and compensatory movement patterns;
3. normalization of the facial-body schema and body image; and.
4. restoration and improvement of recognition and expression in various social contexts.

**Figure 9:**
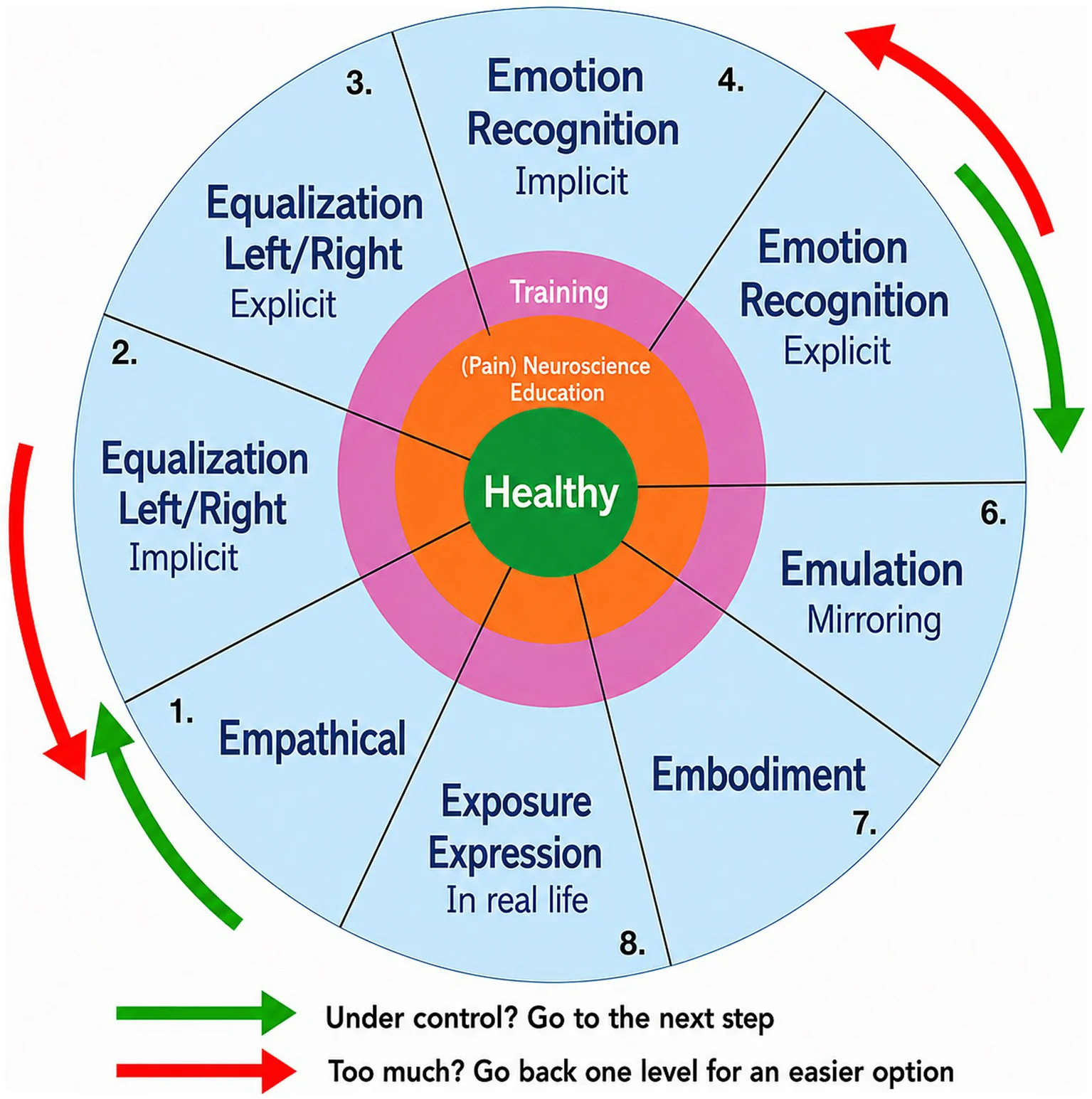
The 8-E Model illustrate a proposed method for the staged rehabilitation of patients suffering from peripheral facial palsy. The schematic overview delineates the eight sequential training stages: Firstly, empathic observation and implicit laterality normalization; secondly, explicit motor imagery, emotion recognition and expression; thirdly, action observation and embodiment; and finally, graded real-life exposure. The circular arrangement is indicative of the graded nature of the program. Patients advance in a clockwise direction only once a stage can be completed without significant anxiety or avoidance. Patients may temporarily step back to a lower level in the event of threat or exacerbation of symptoms.

An overview of the 8E model is shown in Figure 9.

### The 8-E model: staged brain training of peripheral facial palsy

The eight phases are designed to be completed sequentially; however, there is room for clinical flexibility based on the findings of the initial assessment. Advancement to the subsequent phase is contingent upon the completion of the preceding phase in an anxiety-free, avoidance-free, and emotionally stable manner. The model’s general structure mirrors the progression of the GMI, which begins with the implicit motor representation (i.e., the assessment of laterality). It then progresses to the explicit motor imagery stage, followed by active movement execution. Ultimately, the model culminates in contextual generalization.

#### Stage 1: Empathy—observation and visualization of facial motor activity

Motor imagery is a complex neurocognitive process that begins with implicit planning and anticipation of movement prior to any motor execution. In patients diagnosed with PFP, the disruption of efference copy signals and the alteration of expected somatosensory feedback distort this anticipatory motor process, thereby contributing to the maintenance of learned non-use and the perpetuation of body schema disturbance (90, 91). In Stage 1, Imagined Facial Movement (IFM) and Observed Facial Movement (OFM) are systematically introduced. The clinician assists the patient in constructing a hierarchical inventory of facial movements, derived from the laterality judgment and emotion recognition assessment. This inventory is then ordered from least to most threatening or difficult. The patient is instructed to observe these movements performed by a model (OFM) in a passive manner, with the stipulation that they do not experience anxiety, avoidance, or emotional activation during the observation. This stage directly addresses the body schema deficit by reactivating dormant motor representations through safe, non-demanding visuomotor engagement.

#### Stage 2: Equalization (implicit)—implicit laterality judgement training

In implicit Left/Right judgment training, patients are required to swiftly ascertain whether the facial photographs displaying facial movements or expressions exhibit a left or right bias. It is imperative that this process be executed without the conscious execution of any motor actions. This task has been shown to activate the same neural circuits involved in motor planning, including the premotor cortex, the supplementary motor area, the inferior parietal lobe, and the cerebellum (101, 102). This activation occurs through a process of implicit motor simulation. In PFP, the assessment of laterality for the paralyzed half of the face is typically slower and less accurate than for the unaffected side. This finding indicates an impaired motor map in the cerebral cortex on the affected side (101). Systematic laterality training has been demonstrated to normalize these neural representations through gradual exposure with multiple repetitions. The predictive coding framework provides further context for this mechanism: the central nervous system actively generates motor predictions that are compared with incoming sensory information; lateralization training recalibrates these predictive templates toward a symmetrical face-body schema (90, 91).

#### Stage 3: Equalization (explicit)—explicit motor visualization training

**Figure 10:**
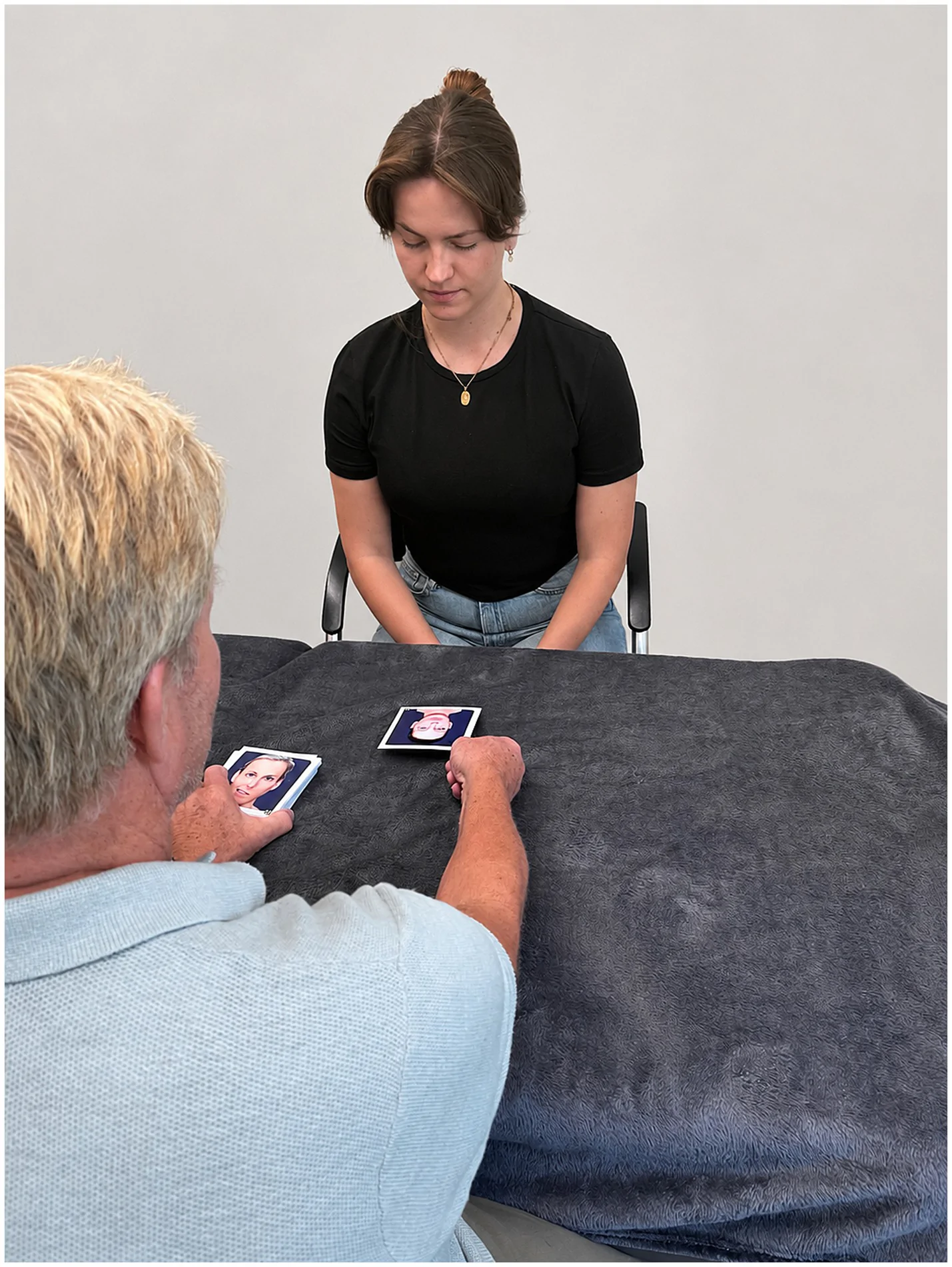
Explicit equalizing (left/right) training. During explicit laterality training, patients identify whether facial images represent the left or right side of the face. This task engages motor imagery processes and promotes activation of facial motor representations without requiring actual movement of the affected muscles. Written informed consent was obtained from the individual(s) for the publication of these identifiable images in this article.

This phase builds on the implicit foundation established in Phase 2. It introduces explicit motor visualization techniques. Patients are instructed to consciously and deliberately imagine performing specific facial movements, particularly those that were limited or absent during the baseline assessment, first on the less affected side and then on the paralyzed side of the face (Figure 10). Explicit motor visualization can be performed in two modes: the visual-internal mode, which employs a kinesthetic, first-person perspective, and the visual-external mode, which employs a third-person perspective. Research has indicated that the kinesthetic modality activates the primary motor cortex and supplementary motor areas with greater reliability (100). In the context of PFP, this phase is of particular importance for restoring voluntary control over paralyzed muscles prior to physical movement, thereby reducing the risk of activating compensatory synkinetic patterns. The gradual progression from simple, symmetrical facial movements to complex, asymmetrical, emotionally charged expressions ensures that the rehabilitation of the motor map in the cerebral cortex proceeds without triggering threat-based avoidance responses.

#### Stage 4: Emotion recognition (implicit)—implicit facial emotion recognition training

Phase 4 addresses the deficit in embodied simulation that is characteristic of PFP. The recalibration of the patient’s internal facial-motor simulation system is achieved through repeated passive exposure to and implicit recognition of basic emotional facial expressions, utilizing standardized photographic stimuli such as MYFaceTraining (MFT) cards. The implicit nature of this phase (recognition without conscious motor execution) ensures that the retraining of the emotional simulation cycle takes place prior to any request for overt emotional facial expressions. Many patients with PFP perceive these expressions as threatening due to the visible asymmetry of their expressive movements. The six basic emotions (joy, sadness, anger, disgust, fear, and surprise) function as stimuli. The training sequence commences with the emotions that are most readily identifiable and progresses sequentially to those that present the most significant processing challenges. In accordance with the embodied simulation framework, both accuracy and reaction time are measured as performance indicators.

#### Stage 5: Emotion expression (explicit)—guided facial emotional expression

In phase 5, patients are guided to consciously and deliberately produce facial expressions representing the six fundamental emotions, commencing with those that are most readily voluntary on the affected side and progressing to expressions that demand the utilization of the most severely paralyzed muscle groups. The therapist provides structured, step-by-step instructions, verbal cues, and—where necessary—gentle tactile support to activate the targeted muscle groups. This phase directly bridges the gap between implicit motor imagery and the actual execution of movements, a transition that is neurologically crucial. The voluntary execution of emotionally driven facial movements engages the supplementary motor area and the limbic-motor integration pathways in a way that is qualitatively different from purely volitional, mechanical movements (93, 98). For patients with a significant disturbance of (facial) body image or anxiety regarding appearance, training in gradations of emotional expression is further embedded within an acceptance-based framework. This framework recognizes that expression does not need to be symmetrical to be therapeutically effective or socially communicative.

#### Stage 6: Emulation—action observation and mirror-based feedback

Emulation is defined as the precise observation and attempted replication of facial movements and expressions performed by another person—the clinician, a video model, or the patient’s own reflection in a bilateral mirror. Action observation activates overlapping fronto-parietal neural networks, including the inferior frontal gyrus, premotor cortex, and inferior parietal lobule. This results in a motor resonance effect that primes the observer’s motor system for subsequent execution (101, 102). The application of mirror visual feedback, otherwise known as mirror therapy, has been demonstrated to create the visual illusion of symmetrical facial movement. This technique has shown clinical efficacy in reducing synkinesis and improving voluntary facial movement in patients diagnosed with PFP. This improvement is likely the result of the artificial normalization of visual feedback to the motor system (103). The emulation stage operationalizes these mechanisms through a graded progression from simple unilateral movement observation to complex bilateral emotional expression observation and replication. Explicit motor imagery has been shown to activate additional subcortical structures, including the basal ganglia and cerebellum, in a manner analogous to actual movement. This process represents a neurologically more demanding process than action observation alone (101).

#### Stage 7: Embodiment—whole-body emotional expression and mind–body integration

Emotional states are not exclusively expressed through facial expressions; other components of nonverbal communication, such as posture, gestures, gaze, and whole-body movements, play a significant role in emotional expression (98). Phase 7 extends the focus of rehabilitation beyond the face to include whole-body emotional expression. This approach addresses the tendency of patients with PFP to develop general physical withdrawal and postural restriction as secondary consequences of facial avoidance behavior. The integration of principles of external attention focus and motor relearning is a central tenet of this approach, wherein the therapist directs the patient’s attention toward the communicative effect of movement on an observer, as opposed to the intrinsic characteristics of the movement itself. This approach has been demonstrated to enhance motor learning by reducing self-monitoring and threat-based attention to the paralyzed facial side (90, 91). The integration of whole-body emotional expression into the rehabilitation framework is further substantiated by evidence from research on embodied cognition, which suggests bidirectional influences between body posture, movement, and affective state.

#### Stage 8: Exposure in real-life contexts—graded *in vivo* socialization and functional generalization

The final stage of the 8-E Model is the translational component, which involves the structured transfer of rehabilitated motor, perceptual, and emotional competencies into the patient’s actual social and occupational environments. The approach of graded *in vivo* exposure is founded on the principles of exposure-based cognitive-behavioral therapy. In this approach, the clinician and patient engage in a collaborative process to construct a hierarchy of social situations, with these situations ordered by their anticipated level of facial self-consciousness or avoidance. The deliberate deployment of facial expressions, eye contact, and emotional communication is first practiced in low-demand contexts (e.g., familiar interpersonal dyadic interactions) and subsequently in higher-demand situations (e.g., group social interactions, professional contexts, video communication). This stage is designed to directly target self-efficacy and functional independence, which are considered the ultimate rehabilitation goals. In addition, it simultaneously addresses the body image and social appearance anxiety dimensions of PFP that conventional neuromuscular programs do not systematically address (40, 92).

### Progression protocol for the 8-E model in peripheral facial palsy

Supplementary Table 3 presents the clinical progression protocol for the 8-E Model in PFP. The fundamental progression criterion throughout is the absence of anxiety, avoidance, or emotional distress. Clinicians are advised to maintain vigilance for indications of threat activation, encompassing postural withdrawal, evasion of mirrored reflection, or emotional distress. These signals necessitate a reduction in task demand or a reversion to the preceding stage. The pain absence progression criterion, utilized in GMI frameworks for chronic pain, is substituted by the criterion of emotional safety, which reflects the primary psycho-social rehabilitation goals in PFP.

This progression scheme reflects a theoretically derived, clinically informed proposal and should be understood as a starting point for future empirical validation rather than a prescriptive, evidence-based protocol.

### Additional approaches to the 8E model

Within the 8-E model, various forms of treatment approaches can be systematically integrated as therapeutic exercises to promote the principle of Cross Modality Plasticity Training systematic variation and combining of inputs from different sensory modalities (for example touch, vision, and audition) in order to drive adaptive reorganization of shared neural networks and enhance integration between these senses (104).

For example, (i) *two-point discrimination* can be transformed from a purely diagnostic measurement into a step-by-step training task, in which patients practice distinguishing distances, locations, and the temporal progression of tactile stimuli on the paralyzed and non-paralyzed halves of the face. By gradually increasing the difficulty of the task in terms of distance, localization, and speed, such exercises can improve somatosensory acuity and contribute to the refinement of facial representation, rather than merely documenting its impairment.

**Figure 11:**
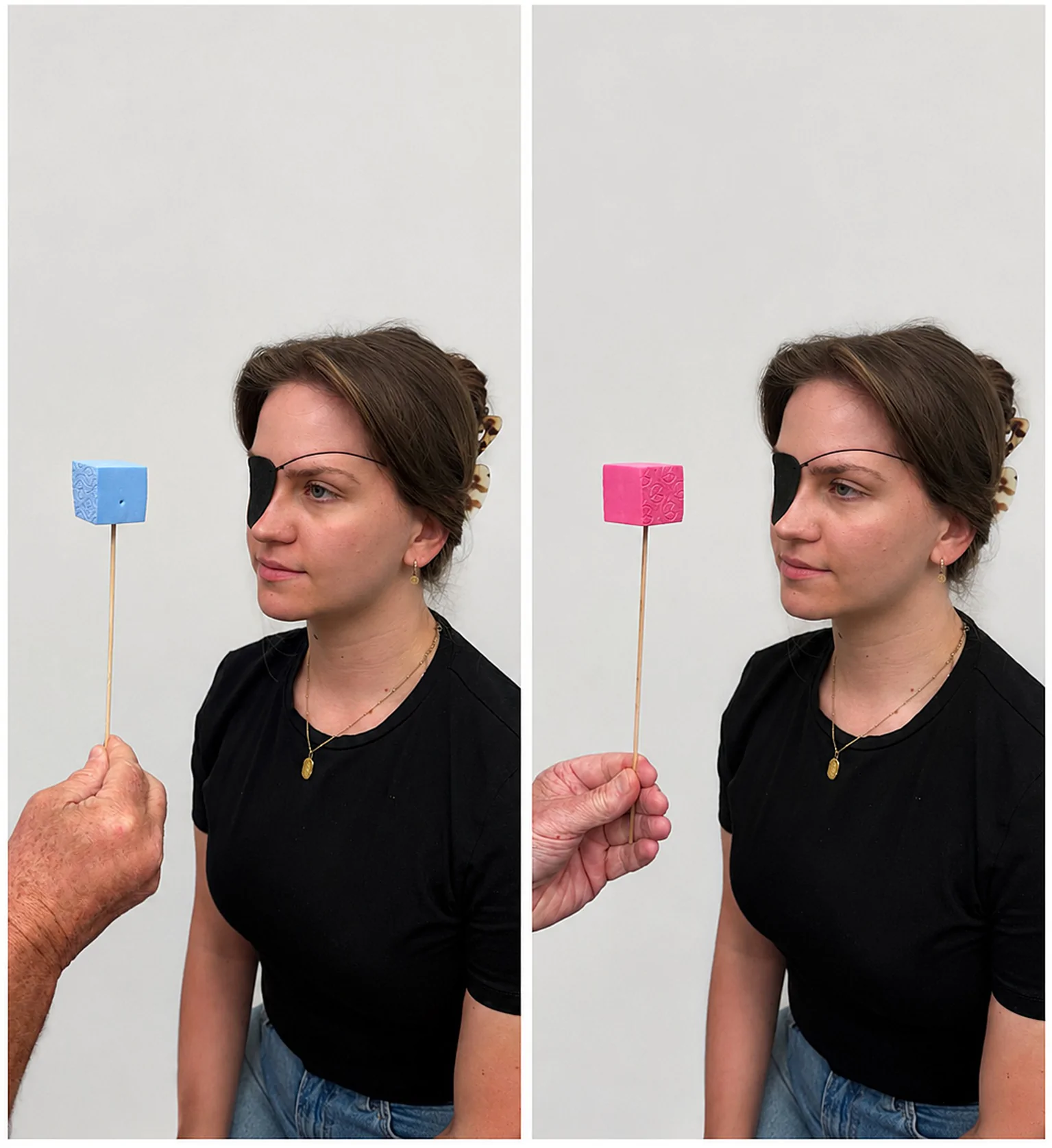
Color-modulated trigeminal blink reflex training. Blue and red visual cues are used therapeutically to modify the perceived threat value of blink-reflex-evoking stimuli. This approach aims to promote habituation, reduce protective responses, and improve sensorimotor processing during rehabilitation. Written informed consent was obtained from the individual(s) for the publication of these identifiable images in this article.

A similar principle applies to neurophysiological tests such as the (ii) *trigeminal blink reflex (TBR).* Instead of using a uniform, purely technical stimulus, the reflex pathway can be activated with behaviorally salient signals, such as colored light blocks (e.g., blue versus red) that are associated with different response requirements or emotional meanings. Experimental research in pain neuroscience, for example by Moseley and Arntz (105), has shown that both temperature and color can influence perceived threat and physiological responses, indicating that seemingly neutral sensory parameters can alter how a stimulus is processed by the central nervous system. In line with this, variations in color, intensity, and predictability of blink-reflex-evoking stimuli could be systematically manipulated to train threat assessment, habituation, and sensorimotor gating, thereby embedding the assessment of the blink reflex within a broader, exposure-based framework (Figure 11).

(iii) *The Hearing Acuity Tests (HAT)* can similarly be adapted into therapeutic formats. A standard hearing acuity test can be expanded into a structured series of exercises in which the frequency of the stimulus, the spatial position (e.g., ipsilateral versus contralateral to the paralysis), and the temporal dynamics (static versus moving sound sources) are systematically varied. For facial palsy patients with hyperacusis or sound intolerance related to facial nerve impairment, such stepwise auditory tasks can help in re-calibrating sound tolerance and integrating auditory input with facial motor control. Although this article does not go into detail on all these protocol variations, the overarching principle is that sensory and neurophysiological clinical tests, as described in the Assessment section, are not merely measurement tools but can also be consciously employed as multimodal training elements within a comprehensive, brain-based rehabilitation program.

In contrast to the preceding sensory-based procedures, which primarily target perceptual and sensorimotor processing, the Pre Lesion Experience using Autobiographical Memory (PEAM) focuses on higher-order cognitive and memory functions. This represents a valuable additional strategy for patients with facial paresis.

### Pre-lesion experience using autobiographical memory (PEAM)

Pre-lesion experience using autobiographical memory (PEAM) is a structured clinical interview method in which the clinician systematically elicits vivid autobiographical memories from before the onset of facial paresis that were closely associated with specific facial expressions and social situations, in order to reconstruct the patient’s pre-lesion facial motor–emotional repertoire and use it as an individual reference framework for assessment and rehabilitation (106, 107).

Assessing pre-lesion experience is well established in neuropsychology as a means of identifying amnesia and changes in self-identity after acquired brain injury. In the context of facial palsy, autobiographical memory therefore could be used as a primary tool during the initial assessment phase. By applying the PEAM approach described by Kuttenreich et al. (107), the therapist systematically explores personally meaningful situations in which the patient’s face was highly expressive prior to the lesion. Through this exploration, the therapist gains a detailed impression of the patient’s habitual facial motor repertoire, its emotional meaning, and the social contexts in which these expressions were embedded, with the goal of defining realistic, individualized motor and emotional targets for subsequent rehabilitation.

Autobiographical memories can be systematically elicited from patients with facial palsy by means of a semi-structured conversational interview. This involves linking specific or complex facial muscle activity to remembered situations and movements; for example, activity of the forehead (m. frontalis) when reacting with surprise to unexpected costs, or smiling (m. zygomaticus) when recalling the last family event (107).

### Systematic PEAM interview to identify clinically useful autobiographical memories

When applying PEAM in patients with facial palsy, the clinician can follow a brief, structured interview sequence:

- *Identify personally meaningful pre-lesion situations*. Ask the patient to recall specific events that occurred before the onset of facial palsy and that were emotionally salient and socially relevant (e.g., a family celebration, a professional presentation, a conflict situation, or a surprising piece of news). Encourage the patient to choose memories in which the face played an important role in communicating emotion or intention rather than neutral, routine events.
- *Link each situation to concrete facial actions*. For each selected event, invite the patient to describe as precisely as possible how the face felt and moved during that situation (for example, “my eyebrows lifted in surprise,” “I smiled broadly,” “my lips were pressed together”). Where possible, the clinician translates these descriptions into specific muscles or muscle groups (for example, *M. frontalis* for eyebrow elevation, M. zygomaticus major for smiling, *M. orbicularis* oris for lip closure) and documents them using standard anatomical terminology.
- *Characterize emotional valence and social context*. The clinician asks which discrete emotion was predominant (for example, joy, sadness, anger, fear, disgust, or surprise) and how intense it was on a simple rating scale (e.g., 0–10). In addition, the social context (who was present, where the interaction took place, and why the expression was important) and sensory impressions (such as information about temperature, sounds or voices, physical contact, odors, etc.) is briefly described, as this information will support the later use of the memory as a meaningful cue during training.
- *Select PEAM targets for assessment and rehabilitation*. From the pool of elicited memories, the clinician selects a limited number of prototypical situations that (a) are highly meaningful to the patient, (b) involve clearly defined facial actions, and (c) are feasible to train within the rehabilitation setting. These PEAM targets are then used both as reference points during baseline assessment (for example, asking the patient to “re-enact” the remembered expression) and as individually tailored goals during motor imagery, mirror therapy, or emotion-expression exercises.

### Rehabilitation strategies PEAM

The utilization of autobiographical memories in a therapeutic capacity has been posited for patients afflicted with both central and peripheral facial palsy (107–111). However, it is imperative to draw a clear distinction between these two conditions. In cases of central facial palsy, the primary objective is to restore motor function to the greatest extent possible, as previously observed prior to the onset of the lesion. In the context of peripheral facial palsy, this principle finds application exclusively in the early phases of recovery. Its application to the chronic post-paralytic phase with synkinesis is not possible.

Peripheral facial palsy is known to progress through three phases of recovery: complete paralysis, partial paresis, and optimal recovery. It should be noted that not all patients go through every phase, but rehabilitation is usually tailored to the current phase as described here (112, 113). The same phasing phenomenon has been observed in relation to motor imagery and the utilization of autobiographical memories. In central facial palsy, and in the paralysis and early paresis phases of peripheral facial palsy, motor experiences from before the lesion can be explicitly used as therapeutic tools: the patient is encouraged to recall their original, pre-lesion facial expressions in personally meaningful situations and to mentally practice them.

As movement gradually returns (paresis phase), the complexity of the PEAM can be increased. A possible approach is to modify the intensity of emotional experience. In natural contexts, emotions may be expressed with varying degrees of intensity, often depending on the situational context. For example, surprise at receiving a salary increase may be pronounced depending on whether the individual had anticipated it beforehand. Similarly, the intensity of a smile may differ depending on whether it reflects genuine joy, serves merely as a gesture of gratitude, or is used as a form of greeting. Emotional expression may also vary according to social norms, expectations, and culture; for instance, grief following the death of a family member is allowed to be expressed openly in private settings, whereas it is more likely to be suppressed in professional situations.

**Figure 12:**
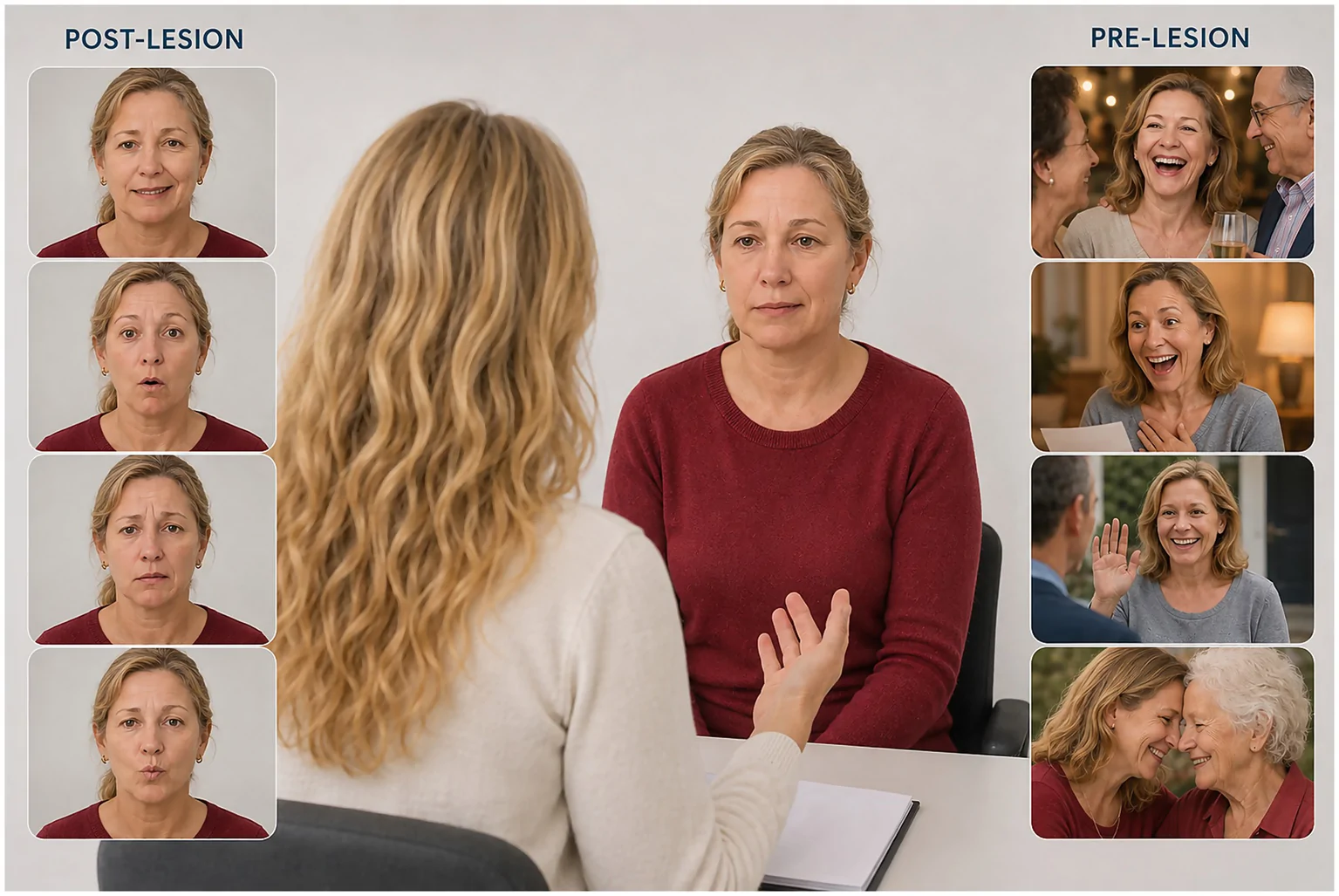
Overview of the pre-lesion experience using autobiographical memory (PEAM) approach. Clinically meaningful pre-lesion autobiographical memories are explored and linked to specific facial expressions. These memories subsequently serve as individualized targets for motor imagery and the development of improved post-lesion facial motor patterns.

Once patients enter post-paralytic phase of peripheral facial palsy and develop synkinesis, further adjustments are required. This phase is frequently characterized by the presence of additional unwanted movements (synkinesis) and other maladaptive movement patterns (see Introduction). It is important to note that pre-lesion motor imagery reinforced at this stage, can stabilize former coordination pattern, which is now dysfunctional. To reduce synkinesis, it is necessary to design, implement, and consolidate new, more selective movement patterns that minimize or eliminate involuntary co-movements. At this stage, motor imagery can once again be incorporated into therapy; however, unlike earlier phases, it is no longer based on pre-lesion movement experiences. Instead, a novel, future-oriented scenario is collaboratively developed with the patient, in which motor imagery with a specific emotional facial expression is systematically associated with a newly established movement pattern. This process requires a detailed and explicit analysis of the individual synkinetic movement patterns previously to identify maladaptive muscle co-activations and their functional impact. The utilization of explicit motor imagery may represent a particularly promising method for the planning, programming, and internalization of these novel patterns. Patients are facilitated in the construction of new post-lesion autobiographical memories, which are associated with enhanced expressions without synkinesis. Repeated practice within emotionally meaningful contexts may further support motor relearning and enhance the generalization of newly acquired movement patterns to daily life situations. These new memories can then be used as meaningful cues in therapy, reinforcing a more flexible, symmetrical, and socially effective facial expression (Figure 12).

### Clinical example: the use of pre- and post-lesion experience using autobiographical memory in different recovery phases of peripheral facial palsy

#### Early paresis phase—the utilization of autobiographical memories from prior to the lesion

A 42-year-old female patient is admitted with idiopathic right-sided peripheral facial paresis, 3 weeks following the onset of symptoms. The patient is in the early paresis phase, which is characterized by weak but visible voluntary movement of the zygomaticus major muscle, and the absence of synkinesis. During the PEAM session, the therapist invites the patient to recall a particularly positive event from prior to the lesion, emphasizing the central role played by her facial expression. She then goes on to describe the birthday party of her son, at which she was present, and states that she “smiled broadly” when he opened his present. The therapist then links this memory to the activation of the zygomaticus major and orbicularis oculi on both sides, noting this as *“Birthday party, broad smile, zygomaticus major / orbicularis oculi.”*

In the course of therapeutic intervention, the patient is firstly requested to recollect the birthday scene with maximum vividness (i.e., the individuals present, the utterances made, and the emotional state experienced), and subsequently to envision herself emulating the aforementioned broad, symmetrical smile whilst mentally rehearsing the kinesthetic sensations experienced in the cheeks and the periorbital region. If needed, the patient can also move her face with her hands and mimic the movement for a deeper sensation. This autobiographical memory from before the lesion serves as a concrete, emotionally meaningful motor goal during both motor imagery and gradual active smile training.

#### The post-paralytic synkinesis phase is characterized by the adoption of novel movement patterns and the persistence of post-lesion memories

In the post-paralytic synkinesis phase, there is a transition to new movement patterns and memories from after the lesion. Following a period of 8 months, the patient continues to manifest clear post-paralytic synkinesis. Specifically, each time she attempts to smile, the eye on the affected side closes involuntarily. Continuing to practice the “old” broad smile in the same way would reinforce the associated eye closure. Consequently, a new movement goal is established: a smile with minimal co-contraction of the eyes. The focus of motor visualization and mirror training has shifted towards this “new smile pattern,” with an explicit emphasis on maintaining relaxed eye regions.

Once the patient can reliably perform this smile with reduced synkinesis, the therapist will help her anchor it in a new autobiographical memory post-lesion. For instance, she details a recent occurrence in which she experienced authentic joy and deliberately employed the “new smile” during a brief exchange at her place of employment. This post-lesion memory (e.g., “first successful work conversation—controlled smile—reduced eye closure”) is then used as a new PEAM-like cue for ongoing visualization and practice, with the aim of supporting the consolidation of a more adaptive, synkinesis-free expression.

### Practical guidelines for using PEAM in peripheral facial palsy

In clinical practice, PEAM should be administered with meticulous attention to the phase of recovery.

- Firstly, the utilization of pre-lesion autobiographical memories is primarily recommended in cases of central facial palsy and the paralysis or early paresis phase of peripheral facial palsy. In such instances, the primary objective is to reactivate the original facial expression pattern.
- Secondly, once post-paralytic synkinesis has emerged, the focus should be shifted towards the design of new, selective movement patterns.
- Thirdly, support should be given to patients in the construction of new post-lesion autobiographical memories that are explicitly linked to these improved, synkinesis-reduced expressions. These memories should then be used as cues for ongoing motor imagery and practice.
- Finally, it is essential to closely monitor patients’ attentional focus on the face and explicitly discuss the potential risks of hyperfocus. This should be balanced with careful observation of movement and the protection of patients’ psychosocial well-being.

## Conclusion

- The management of peripheral facial palsy is evolving from a predominantly motor-oriented approach toward a more comprehensive understanding of sensorimotor, perceptual, and psychosocial processes.
- Emerging evidence indicates that altered somatosensory processing, body awareness, and body representation may contribute to persistent symptoms and functional limitations, although the causal and functional relevance of these findings requires further clarification.
- Consequently, assessment and rehabilitation should extend beyond facial muscle performance and include the evaluation of somatosensory and neurocognitive factors. The proposed 8-E model offers a structured framework for integrating these concepts into clinical practice, although its collective efficacy as an integrated model remains to be empirically validated.
- Incorporating targeted somatosensory training may enhance sensorimotor integration and support more individualized rehabilitation strategies. Further research is required to establish the effectiveness of these approaches and to determine their contribution to long-term recovery and quality of life in individuals with peripheral facial palsy.

## Footnotes

1
https://www.myfacetraining.com
