# Supplementary material for: Beyond motor recovery: emerging perspectives in the management of peripheral facial palsy—a narrative review
Source: Front Neurol. 2026 Sep 7;17:1915135. doi: 10.3389/fneur.2026.1915135 (PMC13595365; doi:10.3389/fneur.2026.1915135)
Supplement: Supplementary file 1 [file Table_1.docx]

**Table 1.** Overview of assessment studies. Search Strategy **Facial palsy (A) and Assessment (B)** (n = 11).

| **Author (year)** | **Content / brief description** | **Main category** | **Study design** | **Level of evidence** | **Comment** |
| --- | --- | --- | --- | --- | --- |
| Bate et al., 2013 (37) | Facial expression recognition, mental imagery of emotional expressions, facial identity processing, lower-level vision, and object recognition were assessed in six individuals with Möbius sequence.  Findings provide evidence against embodied simulation theories of emotional expression processing. | (Facial) emotion recognition | Case series — cross-sectional | Level IV | No control group; very small sample size; findings cannot be generalized. |
| Bogart, 2020 (42) | Adults with congenital or acquired facial palsy completed validated survey measures of emotional clarity, attachment security, stigma, anxiety, and depression.  Individuals with facial palsy - particularly those with acquired onset — are at heightened risk of socioemotional difficulties. Results suggest a congenital advantage in socioemotional adaptation. | Socioemotional functioning | Cohort study — cross-sectional | Level III | No healthy control group; cross-sectional design limits causal inference. |
| Carneiro Pereira & Vannuscorps, 2026 (43) | The necessity of motor simulation for efficient and automatic facial expression recognition was investigated using a dual-task paradigm in individuals with congenital facial palsy (Möbius syndrome).  Automatic and efficient facial expression recognition can occur independently of motor simulation, challenging simulation-based accounts of social perception. | (Facial) emotion recognition and motor simulation | Cross-sectional group comparison with experimental task | Level III | Includes a healthy control group; experimental design is well-controlled. |
| De Carvalho et al., 2019 (39) | The impact of facial palsy and its treatment with botulinum toxin on body image was examined using the Body Image Quality of Life Inventory (BIQLI) before treatment and at 15 and 180 days post-intervention.  Botulinum toxin treatment significantly improved body image and psychosocial well-being, although recovery across all domains was incomplete. | Body image | Prospective cohort study | Level III | No long-term follow-up beyond 180 days; absence of a control condition. |
| Dring et al., 2025 (40) | Social appearance anxiety, general social anxiety, fear of negative evaluation, and appearance-related safety behaviors were assessed in individuals with facial palsy using a multi-dimensional self-report battery (SAAS, SIAS-6/SPS-6, BFNE, BICSI-AF, PHQ-8, FDI-PF).  Individuals with facial palsy reported significantly elevated scores across all psychosocial measures compared to controls; fear of negative evaluation and appearance-fixing behaviors were the primary predictors of social appearance anxiety. | Emotional/psychological processing, body image, and facial function | Cross-sectional survey design | Level III | Multidimensional assessment capturing cognitive, emotional, and body-image dimensions; cross-sectional design limits causal interpretation. |
| Gil-Martínez et al., 2021 (41) | Muscle activation was assessed using surface EMG of key facial muscles during standardized facial gestures, performed with and without mirror visual biofeedback (Specular Face software). The Facial Disability Index (FDI) served as the complementary patient-reported outcome measure.  Computerized mirror therapy demonstrated clinically meaningful reductions in synkinesis and improvements in agonist activation in most participants. | Muscle activation and facial function | Case series — within-subject comparison (with vs. without software) | Level IV | Proof-of-concept only; no longitudinal follow-up or patient-reported outcome data beyond the immediate session. |
| Japee et al., 2023 (44) | Facial emotion perception was assessed in congenital facial palsy using a multi-layered psychophysical battery, including static and dynamic recognition tasks with morph continua and variable emotional intensity, multiple perceptual control tasks, and fMRI in a subset of participants.  Selectively elevated facial emotion detection thresholds were observed in facial palsy, while non-emotional and body-related perception remained largely intact; behavioral deficits were linked to altered neural activity in emotion- and face-sensitive regions. | Facial emotion perception | Quasi-experimental, cross-sectional | Level III | fMRI subsample limits generalizability of neuroimaging findings to the clinical context. |
| Kuttenreich et al., 2022 (45) | Facial emotion recognition (*Myfacetraining Program*) and auditory emotion recognition (*Montreals Affective Voices*) was measured in acquired, unilateral, chronic peripheral facial palsy with synkinesis. Self-assessment was also studied (questionnaires).  Facial and auditory emotion recognition remained intact. Impairments (facial and auditory)  were observed only in a few patients with marked facial asymmetry  and severe facial synkinesis. | Facial and auditory emotion recognition and self-assessment in facial emotion recognition | Controlled prospective cross-sectional study | Level III | Healthy control group; also measurement of auditory emotion recognition for distinction between modality-specific and cross-modal performance |
| Nguyen & Coulson, 2025 (38) | A mixed-methods design was employed, combining the Facial Disability Index (FDI), the Mirror Gazing Cognition and Affect Rating Scale (MG-CARS), and a custom quality-of-life questionnaire with open-ended survey responses analyzed thematically, to assess the psychosocial burden of pregnancy-associated Bell's palsy.  Pregnancy-associated Bell's palsy was associated with substantial impacts on appearance-related distress, social functioning, and quality of life. | Quality of life and facial function | Cross-sectional mixed-methods study | Level III | No healthy control group; small sample size (n = 17). |
| Storbeck et al., 2019 (46) | Emotion recognition was assessed longitudinally in acute Bell's palsy using a computerized test battery combining multiple psychophysical EMO tasks (composite faces, dynamic videos, visual search, DANVA) with accuracy and reaction time outcomes. A control task (FACE task) separated general face perception from emotion-specific processing. Clinician-rated facial function (FNGS 2.0) and patient-reported measures (FaCE, HADS, SF-12) were included.  Facial emotion recognition accuracy was preserved but reaction times were significantly delayed, indicating subtle processing deficits attributable to disrupted embodied simulation. | Facial emotion recognition and objective/subjective deficits in facial palsy | Controlled prospective cohort study | Level II–III | Healthy control group; multiple measurement time points; validated objective and subjective outcome measures; statistical modelling applied. |
| Volk et al., 2022 (19) | Somatosensory function was profiled using the DFNS Quantitative Sensory Testing (QST) protocol applied to the paretic side, contralateral side, and a distal control site. Results were expressed as z-scores relative to normative data and complemented by patient-reported outcomes (FaCE facial comfort subscale, SF-36 pain domain).  Somatosensory function was largely preserved across all QST parameters, with no clinically relevant side-to-side differences, despite patients' subjective sensory complaints. | Sensory function and quality of life | Prospective observational — cross-sectional | Level III | Clinically relevant paradigm, given that facial sensation is primarily mediated by the trigeminal nerve rather than the facial nerve; findings support a cortical mismatch hypothesis. |
