# Supplementary material for: Beyond motor recovery: emerging perspectives in the management of peripheral facial palsy—a narrative review
Source: Front Neurol. 2026 Sep 7;17:1915135. doi: 10.3389/fneur.2026.1915135 (PMC13595365; doi:10.3389/fneur.2026.1915135)
Supplement: Supplementary file 2 [file Table_2.docx]

**Table 2**. Overview of rehabilitation studies. Search strategy **Facial Palsy (A) and Rehabilitation (B)** (n = 6).

| **Author (year)** | **Content / brief description** | **Main category** | **Therapy design** | **Level of evidence** | **Comment** |
| --- | --- | --- | --- | --- | --- |
| Barth et al., 2020 (51) | Mirror Book Therapy (MBT) was added to standard neuromuscular facial rehabilitation in idiopathic facial palsy. The MBT group demonstrated greater improvement in facial function and, most notably, in social participation compared with standard care alone. | Mirror-based visual feedback rehabilitation | Retrospective comparative clinical study (MBT + usual care vs. usual care alone) | Level III | Suggests added value of mirror visual feedback; limited by a small, non-randomized sample and unequal baselines at inclusion. |
| Gil-Martínez et al., 2021 (41) | Specular Face software provided real-time symmetrical facial visual feedback while surface EMG recorded muscle activity. A single-session case series demonstrated immediate reductions in synkinesis and improved agonist activation in most patients. | Computerized mirror therapy combined with EMG biofeedback | Case series — within-subject comparison (with vs. without software) | Level IV | Provides strong mechanistic proof-of-concept; no longitudinal outcome data or patient-reported measures collected beyond the immediate session. |
| Kasahara et al., 2017 (54) | Tape Feedback Therapy applied perioral tape strips to augment tactile somatosensory feedback during eye-closure training. A controlled pilot trial demonstrated a short-term reduction in oro-ocular synkinesis superior to conventional therapy over four weeks. | Somatosensory biofeedback for synkinesis reduction | Small controlled pilot trial (tape feedback vs. conventional therapy, ~4 weeks) | Level II–III | Provides direct evidence that simple tactile feedback can reduce synkinesis; underpowered and limited by short follow-up duration. |
| Bernd et al., 2018 (52) | A newly developed digital biofeedback program magnified subtle facial movements to guide intensive facial muscle training. Software-derived movement scores correlated strongly with House–Brackmann grade; quality of life was markedly reduced across all patients (HB II–IV). | Digital biofeedback platform for guided facial muscle training | Prospective pilot/validation study without control group | Level III–IV | Validates a technological measurement platform; does not establish comparative treatment efficacy. |
| Hotton et al., 2022 (53) | Six condition-specific self-help guides incorporating CBT and ACT principles were evaluated in people with facial palsy. A pre–post quasi-experimental study demonstrated improvements in social functioning and appearance-related distress without measurable change in facial motor function or depression (HADS). | Psychosocial rehabilitation via structured self-help | Pre–post quasi-experimental study (no control group) | Level III | Demonstrates that targeted psychological rehabilitation can improve key patient-reported outcomes independently of motor recovery; authors recommend future RCTs. |
| Zhao et al., 2026 (50) | The trial enrolled 34 patients with acute peripheral facial palsy (≤7 days) and compared standard care plus facial PNF with standard care plus facial PNF combined with mirror therapy. | Mirror therapy combined with PNF | Single-center, randomized controlled trial (RCT) | Level II | Combination significantly improves facial function, shortens recovery time, and exhibits good safety in patients with acute PFP, making it a promising rehabilitation strategy. |
