# Supplementary material for: Beyond motor recovery: emerging perspectives in the management of peripheral facial palsy—a narrative review
Source: Front Neurol. 2026 Sep 7;17:1915135. doi: 10.3389/fneur.2026.1915135 (PMC13595365; doi:10.3389/fneur.2026.1915135)
Supplement: Supplementary file 3 [file Table_3.docx]

**Table 3**. Progression protocol for the 8-E Model in peripheral facial paresis. RT = reaction time.

| **Stage (Task Level)** | **Starting Level** | **Progression (if no anxiety/distress)** | **Clinical Decision Points** |
| --- | --- | --- | --- |
| 1. Empathy Observation of facial activity (left/right) from images | Begin with 10 facial photographs; exposure ≥5 s per image Task: observe and identify left or right facial movement | 15–20 images; faster presentation rate | Anxiety/avoidance? Reduce images and/or pace. No anxiety → proceed to Stage 2 |
| 2. Equalization (Implicit) Left/right laterality judgement (implicit) | Begin with 10–15 facial images Task: rapidly identify left/right facial hemiface; record accuracy and response time | Increase images; add rotated images (90°, 180°) | Anxiety/avoidance? Reduce images or return to Stage 1. Adequate speed and accuracy → proceed to Stage 3 |
| 3. Equalization (Explicit) Explicit motor imagery — left/right movement execution | Begin with 10–15 images; commence on less-affected side Task: consciously imagine performing facial movements | Progress to paretic side; increase to ≥20 images; 50% paretic side | Anxiety/synkinesis? Reduce paretic-side demand to 25% or return to Stage 2 |
| 4. Emotion Recognition (Implicit) Implicit recognition of basic emotional expressions | Begin with most easily recognized emotions Task: identify emotion depicted; record accuracy and RT | Progress to 20% more difficult emotions; increase speed | Avoidance/distress? Remain with accessible emotions; do not advance |
| 5. Emotion Expression (Explicit) Deliberate facial emotional expression | Begin with accessible emotions on less-affected side Task: produce facial expression of target emotion | Progress to more demanding expressions; include paretic hemiface | Synkinesis or distress? Reduce demand; return to Stage 4 if needed |
| 6. Emulation Action observation and mirror-based feedback | Observe model; attempt replication; introduce bilateral mirror Task: replicate observed facial movements as precisely as possible | Increase complexity; include bilateral emotional expressions; live mirror feedback | Distress with mirror reflection? Remove mirror; reintroduce gradually |
| 7. Embodiment Whole-body emotional expression | Integrate facial expression with posture and gesture Task: express emotions through whole-body movement in a safe therapeutic context | Progress to more complex emotional scenarios; introduce context variation | Withdrawal or avoidance? Reduce demand; focus on postural aspects before facial |
| 8. Exposure in Real-Life Contexts Graded in vivo socialization | Begin with low-demand, familiar social contexts (dyadic conversation) Task: deploy facial expression and emotional communication in real-world settings | Progress to higher-demand social contexts; video communication; professional settings | Anxiety/avoidance? Return to lower-demand context; review Stage 7 |
